# PRP4KA, a Putative Spliceosomal Protein Kinase, Is Important for Alternative Splicing and Development in *Arabidopsis thaliana*

**DOI:** 10.1534/genetics.118.301515

**Published:** 2018-10-08

**Authors:** Tatsuo Kanno, Peter Venhuizen, Tuan-Nan Wen, Wen-Dar Lin, Phebe Chiou, Maria Kalyna, Antonius J. M. Matzke, Marjori Matzke

**Affiliations:** * Institute of Plant and Microbial Biology, Academia Sinica, Nangang District, 115 Taipei, Taiwan; † Department of Applied Genetics and Cell Biology, University of Natural Resources and Life Sciences (BOKU), 1190 Vienna, Austria

**Keywords:** alternative splicing, *Arabidopsis thaliana*, protein phosphorylation, PRP4 kinase, SAC3A

## Abstract

Splicing of precursor messenger RNAs (pre-mRNAs) is an essential step in the expression of most eukaryotic genes. Both constitutive splicing and alternative splicing, which produces multiple messenger RNA (mRNA) isoforms from a single primary transcript, are modulated by reversible protein phosphorylation. Although the plant splicing machinery is known to be a target for phosphorylation, the protein kinases involved remain to be fully defined. We report here the identification of pre-mRNA processing 4 (PRP4) KINASE A (PRP4KA) in a forward genetic screen based on an alternatively spliced *GFP* reporter gene in *Arabidopsis thaliana* (*Arabidopsis*). Prp4 kinase is the first spliceosome-associated kinase shown to regulate splicing in fungi and mammals but it has not yet been studied in plants. In the same screen we identified mutants defective in SAC3A, a putative mRNA export factor that is highly coexpressed with PRP4KA in *Arabidopsis*. Whereas the *sac3a* mutants appear normal, the *prp4ka* mutants display a pleiotropic phenotype featuring atypical rosettes, late flowering, tall final stature, reduced branching, and lowered seed set. Analysis of RNA-sequencing data from *prp4ka* and *sac3a* mutants identified widespread and partially overlapping perturbations in alternative splicing in the two mutants. Quantitative phosphoproteomic profiling of a *prp4ka* mutant detected phosphorylation changes in several serine/arginine-rich proteins, which regulate constitutive and alternative splicing, and other splicing-related factors. Tests of PRP4KB, the paralog of PRP4KA, indicated that the two genes are not functionally redundant. The results demonstrate the importance of PRP4KA for alternative splicing and plant phenotype, and suggest that PRP4KA may influence alternative splicing patterns by phosphorylating a subset of splicing regulators.

## Abstract (precis)

Prp4 kinase (Prp4k) is the first spliceosome-associated kinase shown to regulate splicing in fungi and metazoans, but nothing is yet known about its functions in plants. Here, Kanno and Venhuizen *et al.* report...

PRECURSOR messenger RNA (pre-mRNA) splicing, which entails removal of introns and joining of exons, is an essential step in the expression of most eukaryotic genes. Splicing is catalyzed in two consecutive transesterification steps by the spliceosome, a large, dynamic ribonucleoprotein machine located in the nucleus (Will and Lührmann 2011; Matera and Wang 2014; Meyer 2016). In constitutive splicing, the same splice sites are always used, generating a single processed messenger RNA (mRNA) from a given gene. By contrast, alternative splicing involves varying splice-site usage, thus yielding multiple mRNA isoforms from a single primary transcript. Alternative splicing greatly expands transcriptome and proteome diversity. Although rare in *Saccharomyces cerevisiae* (budding yeast) (Gould *et al.* 2016), alternative splicing occurs at low frequency in *Schizosaccharomyces pombe* (fission yeast) (Fair and Pleiss 2017) and is common in plants and metazoans (Nilsen and Graveley 2010; Marquez *et al.* 2012; Naftelberg *et al.* 2015). Major modes of alternative splicing include intron retention (IR), exon skipping (ES), alternative 5′ (donor) splice site, and alternative 3′ (acceptor) splice site. Splicing of exonic introns (exitrons), which are alternatively spliced internal regions of reference protein-coding exons, represents a noncanonical splicing event and occurs in ∼7% of *Arabidopsis* and 4% of human protein-coding genes (Marquez *et al.* 2015; Staiger and Simpson 2015; Sibley *et al.* 2016; Zhang *et al.* 2017). ES is the most frequent mode of alternative splicing in animal cells, whereas it is rarely observed in plants (Marquez *et al.* 2012). IR predominates in plants and is also widespread in animals (Marquez *et al.* 2012; Braunschweig *et al.* 2014). In plants, alternative splicing has important roles in development and in responses to the environment (Staiger and Brown 2013; Filichkin *et al.* 2015; Szakonyi and Duque 2018).

The recognition of alternative splice sites and modulation of splicing events is guided by a splicing code, which involves a complex interplay among *trans*-acting factors, *cis*-acting RNA regulatory elements, and other RNA and chromatin features (Barash *et al.* 2010; Baralle and Baralle 2018). *Trans*-acting splicing factors include serine/arginine-rich (SR) proteins and heterogeneous nuclear ribonucleoproteins (hnRNPs), which respectively bind exonic and intronic *cis*-regulatory elements, which are termed splicing enhancers and silencers (Barta *et al.* 2008; Matera and Wang 2014). Because splicing is coupled to transcription, chromatin structure can influence alternative splicing patterns by influencing the rate of transcription, exon definition, and recruitment of splicing factors through chromatin binding proteins (Naftelberg *et al.* 2015).

Post-translational modifications of splicing proteins (such as phosphorylation, acetylation, ubiquitination, and sumoylation) contribute to the regulation of both constitutive and alternative splicing (Will and Lührmann 2011; Pozzi *et al.* 2017). In particular, reversible phosphorylation of SR proteins and other splicing-related factors has an essential role in splicing (Fluhr 2008; Stamm 2008; Will and Lührmann 2011). SR proteins, which are present in organisms with more complex splicing patterns (fission yeast, plants, and metazoans), feature one or two RNA recognition motifs at their N terminus and an arginine/serine-rich (RS) domain at their C terminus. Phosphorylation/dephosphorylation in the RS domain can alter the ability of SR proteins to interact with other proteins and RNA, which in turn modifies pre-mRNA splicing outcomes (Barta *et al.* 2008; Will and Lührmann 2011). The plant spliceosomal machinery is a major target of phosphorylation, as illustrated by a previous phosphoproteomic investigation in *Arabidopsis thaliana* (*Arabidopsis*), which identified 22 phosphoproteins with a putative role in RNA metabolism. The set of phosphoproteins included 11 out of 18 SR proteins encoded in the *Arabidopsis* genome (de la Fuente van Bentem *et al.* 2006).

In diverse organisms, SR proteins can be phosphorylated by several distinct families of conserved protein kinases (Fluhr 2008; Zhou and Fu 2013). Kinases found previously to be important for phosphorylating SR proteins in *Arabidopsis* include SR protein kinases (de la Fuente van Bentem *et al.* 2006; Rosembert 2017), Cdc2-like or LAMMER-type kinases (Golovkin and Reddy 1999; Savaldi-Goldstein *et al.* 2000), and mitogen-activated protein kinases (Feilner *et al.* 2005; de la Fuente van Bentem *et al.* 2006, 2008).

Pre-mRNA processing 4 (PRP4) kinases, which are dual-specificity kinases (Lehti-Shiu and Shiu 2012), represent another general class of protein kinase involved in phosphorylating SR proteins and other splicing factors (Will and Lührmann 2011; Lützelberger and Käufer 2012). Prp4 kinases are present in all eukaryotes examined except the fungal group Hemiascomycetes, which includes budding yeast. Prp4 kinase was discovered in fission yeast as a temperature-sensitive mutant defective in pre-mRNA splicing at the restrictive temperature (Alahari *et al.* 1993). Although Prp4 kinase is the first kinase shown to regulate pre-mRNA splicing in fungi and mammals (Lützelberger and Käufer 2012), it has not yet been studied for its role in splicing in plants (Lehti-Shiu and Shiu 2012).

We report here the recovery of mutants defective in PRP4 kinase A (PRP4KA) (At3g25840) in a forward genetic screen designed to identify factors that influence splicing of an alternatively spliced *GFP* reporter gene in *Arabidopsis*. In the same screen, we also retrieved mutants impaired in SAC3A (suppressor of actin; Novick *et al.* 1989) (At2g39340), a putative mRNA export factor that is highly coexpressed with PRP4KA in *Arabidopsis*. We describe the phenotypes of *prp4ka* and *sac3a* mutants as well as findings from RNA-sequencing (RNA-seq) analyses to determine differential gene expression and alternative splicing profiles in the two mutants. We present results from a quantitative phosphoproteomic investigation of a *prp4ka* mutant to identify potential substrates of this kinase. Finally, we describe tests of a mutant defective in *PRP4KB* (At1g13350), the paralog of *PRP4KA*, to address possible functional redundancy of the paralogous *PRP4K* genes (Al-Ayoubi *et al.* 2012).

## Materials and Methods

### Plant material

The *Arabidopsis* transgenic T line containing an alternatively spliced *GFP* reporter gene (referred to here as “wild type”) and the *prp4ka and sac3a* mutants generated by ethyl methanesulfonate (EMS) mutagenesis of the T line are in the Col-0 ecotype (Kanno *et al.* 2016, 2017a,b). Seeds of a *prp4kb* transfer-DNA (T-DNA) insertion mutant (SALK_035104C) were provided by the Nottingham *Arabidopsis* Stock Center. The T-DNA is inserted into the middle of the ninth exon. To our knowledge, this is the first report of the *prp4kb* T-DNA insertion mutant, which we will refer to as *prp4kb-1*. The *prp4kb-1* allele appears to be a complete knockout (Supplemental Material, Figure S1). All plants were cultivated under long-day conditions (22–23°, 16 hr light, 8 hr dark).

The terminology used for different plant generations is as follows: The M_2_ generation refers to progeny resulting from self-fertilization (selfing) of the original M_1_ mutant plant grown from seeds treated with EMS. M_1_ progeny are heterozygous for a given mutation. Thus, M_2_ is the first generation when a recessive mutation can be homozygous. Further selfing of the M_2_ plants leads to generations M_3_, M_4_, and so on. Backcrossing an M_2_ plant with the parental wild-type T line produces the BC_1_ generation, which is again heterozygous for the respective mutation. Selfing of BC_1_ plants produces the BC_1_F_2_ generation, 25% of which are again homozygous for the respective mutation. BC_1_F_2_ plants contain fewer EMS-induced mutations than the original M_2_ plant. Further selfing of BC_1_F_2_ plants produces generations BC_1_F_3_, BC_1_F_4_, and so forth. Crossing two strains that are homozygous for different mutations produces the F_1_ generation, which is heterozygous for the two mutations. Selfing an F_1_ plant produces the F_2_ generation, which is segregating the two mutations in a Mendelian manner.

### Forward genetic screen, phenotype analysis, and complementation

The forward genetic screen based on an alternatively spliced *GFP* reporter gene in the wild-type T line has been described previously (Kanno *et al.* 2016, 2017a,b). The mutagen EMS generates almost exclusively G/A to C/T transition mutations (Kim *et al.* 2006). Screening of putative mutants was performed in the M_2_ generation. The *gfw5* and *gfw6* mutants described here were identified by the GFP-weak phenotype of M_2_ seedlings cultivated under sterile conditions on solid Murashige and Skoog medium viewed using a Leica M165FC fluorescence stereomicroscope. The first alleles in the *PRP4KA* gene (At3g25840) and in the *SAC3A* gene (At2g39340) in *gfw5* and *gfw6* mutants, respectively, were identified by next generation mapping (NGM) (James *et al.* 2013). NGM involves sequencing of pooled DNA isolated from at least 50 BC_1_F_2_ seedlings that display a GFP-weak phenotype (Kanno *et al.* 2017a,b). Additional *prp4ka* and *sac3a* alleles were identified by Sanger sequencing of the *PRP4KA* and *SAC3A* genes considered as possible candidates for mutations in unnamed mutants.

Phenotypic analysis of *prp4ka* and *sac3a* mutants (two alleles of each) was performed on the BC_1_F_3_ generation. A total of 12 plants from each genotype (wild-type T line, *prp4ka-2*, *prpk4a-4*, *sac3a-3*, and *sac3a-6* mutants) were grown side by side on soil under long-day conditions (22–23°, 16 hr light, 8 hr dark) and observed during the entire vegetative growth, reproductive phases, and into senescence. The phenotypic characters that were scored included flowering time (time to bolting), rosette diameter, final height of adult plant, number of main and auxiliary stems/branches, seed weight from individual plants, and (in some cases) numbers of siliques per plant and seeds per silique.

For complementation tests, the *prp4ka-4* and *sac3a-6* mutants were transformed with a construct containing either the *PRP4KA* or *SAC3A* wild-type coding sequence under the transcriptional control of the 35S promoter and terminator sequences (Pietrzak *et al.* 1986). The constructs were introduced into the respective mutant plants (BC_1_F_3_ generation) using the floral dip method (Clough and Bent 1998) and *Agrobacterium* binary vector BV-Mp*PAT*ot *Sal*I (Matzke *et al.* 2010), which encodes resistance to phosphinothricin (PPT). T_1_ transformants were selected on solid Murashige and Skoog medium containing 20 µg/ml PPT and 200 µg/ml cefotaxime to destroy agrobacteria. Successful complementation was indicated by a return to an intermediate GFP phenotype (similar to that observed in the wild-type T line) in seedlings growing on solid Murashige and Skoog medium and, in the *prp4ka* mutants, restoration of a wild-type phenotype in soil-grown plants. The presence of the respective *prp4ka-4* and *sac3a-6* mutations in complemented lines was confirmed by Sanger sequencing.

### Western blotting using a GFP antibody

Western blotting to determine levels of GFP protein in the *prp4ka-4* and *sac3a-6* mutants compared to wild-type T line was carried out as described previously (Fu *et al.* 2015; Kanno *et al.* 2016, 2017a,b). Total protein was isolated from 2-week-old seedlings growing on solid Murashige and Skoog medium under a 16 hr light/8 hr dark cycle at 24°. Monoclonal antibodies to GFP were purchased from Roche (catalog no. 11814 460001). For a loading control, a duplicate gel containing the same samples was run and stained with Coomassie brilliant blue.

### Semiquantitative RT-PCR to assess levels of *GFP* RNA splicing variants

Semiquantitative RT-PCR was used to gauge the levels of the three *GFP* RNA splice variants in *prp4ka-4* and *sac3a-6* mutants relative to the wild-type T line following a published protocol (Sasaki *et al.* 2015; Kanno *et al.* 2017a,b). Total RNA was isolated from 2-week-old seedlings of the wild-type T line, the *prp4ka-4* mutant, and the *sac3a-6* mutant (BC_1_F_3_ generation for both mutants) growing on solid Murashige and Skoog medium as described above using a Plant Total RNA Miniprep Kit (GeneMark, Taichung, Taiwan). Primers for *GFP* and actin are listed in Table S1.

### RNA-seq

Total RNA was isolated from 2-week-old seedlings of the wild-type T line, the *prp4ka-4* mutant, and the *sac3a-6* mutant (BC_1_F_3_ generation for both mutants) cultivated on Murashige and Skoog medium as described above. Preparation of libraries and RNA-seq were performed (biological triplicates for each sample) as described previously (Sasaki *et al.* 2015; Kanno *et al.* 2016). Whole-genome resequencing of the *prp4ka-4* and *sac3a-6* mutants was conducted to identify any remaining EMS-induced, second-site mutations that change splice sites. These mutations were then removed from the analysis of alternative splicing.

### RNA-seq analyses for differentially expressed genes and alternative splicing events

#### Differential expression analysis:

To determine differential expression of the *prp4ka* and *sac3a* mutants compared to the wild type, we considered the transcript per million (TPM) estimated with Salmon (version 0.8.0; Patro *et al.* 2017) for the Reference Transcript Dataset for *Arabidopsis* 2 (AtRTD2)-Quantification of Alternatively Spliced Isoforms (QUASI) (AtRTD2-QUASI) annotation (Zhang *et al.* 2017), and used tximport (Soneson *et al.* 2016) to group transcript read counts per gene. Differential genes were determined using edgeR (version 3.18.1; Robinson *et al.* 2010). Genes were considered differentially expressed for a false discovery rate <0.05.

#### Alternative splicing analysis:

Alternative splicing events were generated using SUPPA (Alamancos *et al.* 2015) from the AtRTD2-QUASI reference transcriptome annotation file (Zhang *et al.* 2017). The ES, IR, and exitron events were extracted using variable boundaries, whereas the alternative 3′ and 5′ splicing events (A3 and A5, respectively) were defined with strict boundaries. The percent spliced-in (PSI) inclusion values were calculated based on the transcript TPM quantification. Differential splicing, the ΔPSI, was calculated using the event PSI and Salmon TPM values as input. Events were considered significantly changed for an absolute ΔPSI ≥ 0.1 and a *P*-value of <0.01. Introns with a U12 signature were derived from the analysis performed by Zhang *et al.* (2017).

#### SNP/indel calling:

SNPs and indels were identified using the Genome Analysis Toolkit (GATK) pipeline (Van der Auwera *et al.* 2013). Picard (version 2.10.9, http://broadinstitute.github.io/picard) was used to generate the sequence dictionary for the TAIR10 genome release. Reads were aligned to the TAIR10 genome using BWA-MEM (0.7.16a-r1181; Li 2013), with the added -M flag. The resulting SAM file was converted to BAM format, sorted, and duplicates were marked using Picard tools. The GATK (version 3.8-0-ge9d806836) haplotypeCaller was used to obtain the raw variants and the SelectVariants function was used to extract the SNPs and indels. SNPs were filtered using the following filter expression: “QD < 2.0 || FS > 60.0 || MQ < 40.0 || MQRankSum < −12.5 || ReadPosRankSum < −8.0.” The filter expression for indels was as follows: “QD < 2.0 || FS > 200.0 || ReadPosRankSum < −20.0.”

SNPs and indels were intersected with the AtRTD2 annotated transcripts and the SUPPA events using in-house scripts. Any events with a SNP and/or indel overlapping with either the 5′ splice site or the 3′ splice site were removed from the final output. For the 5′ splice site, the last 3 exonic and the first 10 intronic bases were taken; for the 3′ splice the last 14 intronic bases and the first 3 exonic bases were used.

#### Analysis of alternative introns differentially regulated in *prp4ka* and *sac3a* mutants:

Introns were analyzed per alternative splicing event type, comparing the features of the differentially spliced introns against the introns of the same event type not changed in the mutants. Due to the limited size of the shared and same subgroups, the different types of alternative splicing events were grouped and compared to all events in the *prp4ka* and *sac3a* mutants. Splice-site strengths were evaluated by using position weight matrices (Sheth *et al.* 2006).

#### PRP4KA-dependent first intron splicing:

The differentially regulated IR events in the *prp4ka* mutant were divided into two categories: the first introns of a transcript and all other remaining introns. Splice-site strengths for the first and the remaining introns were evaluated by using position weight matrices (Sheth *et al.* 2006). The degrees of retention (expressed as PSI values) of the first and remaining introns were compared for the wild-type and *prp4ka* genotypes.

### Isobaric tags for relative and absolute quantification analysis

#### Protein preparations and liquid chromatography–mass spectrometry:

Total protein was isolated from 1 g of 2-week-old seedlings growing on solid Murashige and Skoog medium as described above following a previously described protocol (Vélez-Bermúdez *et al.* 2016). Protein treatment, protease digestion, and labeling prior to liquid chromatography (LC)–mass spectrometry (MS) (LC-MS) analysis were performed as described previously (Lan *et al.* 2011) with minor modifications. Protein concentrations were measured using a Pierce 660 nm protein Assay kit (Thermo Scientific). Proteins in 8 M urea, 50 mM Tris-HCl, pH 8.5, were reduced in 10 mM DTT for 1 hr at 37° and Cys were alkylated in 50 mM iodoacetamide at room temperature for 30 min in the dark. The protein solution was then diluted to contain 4 M urea with 50 mM Tris-Cl, pH 8.5; digested with 250 units/ml Benzonase (Sigma-Aldrich, St. Louis, MO) at room temperature for 2 hr; followed by Lys-C (Wako, Osaka, Japan) digestion [1:200 weight by weight (w/w)] at room temperature for 4 hr. The protein solution was further diluted to contain <2 M urea with 50 mM Tris-Cl, pH 8.0, and incubated with modified trypsin (1:50 w/w; Promega, Madison, WI) at 37° overnight. The digested solution was acidified with 10% trifluoroacetic acid, desalted using an Oasis HLB cartridge (Waters Associates, Milford, MA), and dried using a SpeedVac. For phosphoproteome analysis, prior to isobaric tags for relative and absolute quantification (iTRAQ) labeling, phosphopeptides were enriched from digested proteins (3.5 mg) using TiO_2_ affinity chromatography (Titansphere Phos-TiO; GL Sciences) according to the method described by the vendor.

#### Peptide labeling with isobaric tags and fractionation:

Dissolution of dried peptides in dissolution buffer and labeling with iTRAQ reagents (Multiplex kit; AB Sciex) were performed according to the manufacturer’s instructions. Tryptic peptides from two different samples, *prp4ka-4* and the wild-type T line, were labeled with iTRAQ 116 and 117 reagents, respectively. For the phosphoproteome analysis, the *prp4ka-4* and T-line samples were labeled with 114 and 115 reagents, respectively, after the phosphopeptides were enriched using TiO_2_ affinity chromatography. The labeling reactions with iTRAQ reagents were incubated for 1 hr at room temperature. Following the reaction, solutions from different iTRAQ labels were combined and further fractionated on a strong cation-exchange (SCX) (PolySulfoethyl A, 4.6 × 200 mm, 5 µm, 200 Å; PolyLC) HPLC. The SCX chromatography was performed with an initial equilibrium buffer A containing 10 mM KH_2_PO_4_, 25% acetonitrile, pH 2.65, followed by a 0–15% buffer B (1 M KCl in buffer A, pH 2.65) gradient for 20 min, a 15–30% buffer B gradient for 10 min, a 30–50% buffer B gradient for 5 min, a 50–100% buffer B gradient for 1 min, and 100% buffer B for 5 min. The flow rate was 1 ml/min. The chromatography was recorded with absorbance 214 nm UV light. Fractions (0.5 min/fraction) were collected and pooled into 16 final fractions. Fractions were desalted using an Oasis HLB Cartridge (Waters) prior to LC-MS/MS analysis. Enriched phosphopeptide sample was fractionated using hydrophilic interaction liquid chromatography (HILIC) (TSKgel Amide-80 HR, 4.6 × 250 mm, 5 μm; Tosoh). The HILIC was performed in solvent containing acetonitrile and 0.1% trifluoroacetic acid with decreasing acetonitrile gradients: 90–85% in 5 min, 85–60% in 50 min, and 60–0% in 5 min, at flow rate of 0.5 ml/min. Ten fractions were collected for LC-MS/MS analysis.

#### LC-MS/MS analysis:

Peptides in each fraction were redissolved in 0.1% formic acid and the LC-MS/MS was performed using the Q Exactive Mass Spectrometer equipped with the Dionex UltiMate 3000 RSLCnano LC system or the LTQ-Orbitrap Fusion Lumos Mass Spectrometer equipped with the EASY-nLC system. A C18 capillary column (Acclaim PepMap RSLC, 75 μm × 250 mm; Thermo Scientific) was used to separate peptides with a 120-min linear gradient (from 3 to 35%) of solvent B (0.1% formic acid in acetonitrile) at a flow rate of 300 nl/min on the LC system. The MS was operated in the data-dependent mode with the top 10 (Q Exactive) or top 20 (Fusion Lumos) ions (charge states ≥2) for MS/MS analysis following an MS survey scan for each acquisition cycle. The selected ions were isolated in the quadrupole and subsequently fragmented using higher-energy C-trap dissociation (HCD) and then analyzed in the Orbitrap cell. The MS was set as follows on Q Exactive: mass-to-charge ratio range of 380–1800, resolving power of 70,000, automatic gain control (AGC) target of 3*e*^6^, and maximum ion trap (IT) of 30 ms. For the Fusion Lumos Mass Spectrometer, the MS was set as follows: resolving power of 120,000, AGC target of 4*e*^5^, maximum IT of 50 ms. The MS/MS was set as follows on Q Exactive: resolving power of 17,500, AGC target of 1*e*^5^, maximum IT of 200 ms, and HCD collision energy (NCE) of 30%. For the Fusion Lumos Mass Spectrometer, the MS was set as follows: resolution power of 15,000, AGC target of 5*e*^4^, maximum IT of 100 ms, and HCD NCE of 35%. For phosphopeptides, the HCD was set at 30 with 10% stepped NCE on Q Exactive, or at 35% NCE with 5% stepped NCE on the Fusion Lumos Mass Spectrometer.

#### Data analysis for protein identification and quantification:

Peptide identification was performed using the Proteome Discoverer software (version 2.1; Thermo Scientific) with the Sequest HT and Mascot (version 2.5; Matrix Sciences) search engines. MS data were searched against the AtRTD2 translation (Zhang *et al.* 2017) database. Search conditions were set as follows: full trypsin digestion, two maximum missed cleavage allowed, precursor mass tolerance of 10 ppm, fragment mass tolerance of 20 mmu, dynamic modifications of oxidation (M), protein N-terminal acetylation, iTRAQ4plex (Y), static modifications of carbamidomethyl (C), and iTRAQ4plex (N terminus and K). The peptide spectrum matches (PSMs) were validated using the Percolator validator algorithm, which automatically conducted a decoy database search and rescored PSMs using *q*-values and posterior error probabilities. All PSMs were filtered with a *q*-value threshold of 0.05 (5% false discovery rate, FDR) or 0.01 (1% FDR) for proteome or phosphoproteome analysis, respectively. A *q*-value threshold of at least 0.01 was finally used to filter protein FDR for the proteome analysis. For comparative peptide:protein quantification, the ratios (114:115 and 116:117) of iTRAQ reporter ion intensities in MS/MS spectra of PSMs were used to calculate the fold changes between samples.

### Statistical analysis of iTRAQ data

For each phosphoproteome analysis table made by the Proteome Discoverer software (biological replicates 1–3), phosphorylation sites were notated in the form of amino acid:protein:position according to reported peptide sequences, modifications, and master protein accessions. For example: (1) peptide ILSSLSR and modification Phospho [S3(100)] were interpreted as Phospho:S:AT1G01050.P2:24, (2) DEPAEESDGDLGFGLFD and Phospho [S7(100)] were interpreted as Phospho:S:AT1G01100.P2:102:AT4G00810.c1:103:AT5G47700.2:103 because of multiple master proteins, and (3) QSDTSPPPSPASK and Phospho [T/S] were interpreted as S:AT1G01320.1:146/149/153/156|T:AT1G01320.1:148 because of no explicit position of S. In so doing, all records related with the same phosphorylation site in the phosphoproteome analysis tables of biological replicates would result in the same notation, and its normalized abundances in the control and the treatment samples were added up for each replicate. In each phosphoproteome analysis table, log ratios of phosphorylation sites were computed based on the abundance sums and then transferred into Z-scores. In so doing, a phosphorylation site was associated with as many Z-scores as many times of detection in biological replicates. Assuming Z-scores close to zero as for unchanged abundance between the control and the treatment samples, every phosphorylation site with two or more Z-scores was tested for its deviation from “unchanged abundance” by testing the deviation of zero from its Z-scores using a model of the standard normal distribution. Note that the underlying null hypothesis assumes that the abundance of a phosphorylation site was not changed in all three replicates, and a significant *P*-value indicates altered abundance in the mutant samples. A similar method was applied to the proteome analysis tables for detecting proteins with altered abundance in the three replicates. Finally, the two statistical results were joined according to reported phosphorylation–protein relationships.

### Gene ontology classification

Gene ontology (GO) classification of the genes affected in *prp4ka* and *sac3a* mutants and GO term overrepresentation tests were done using PANTHER software tools (Thomas *et al.* 2003) (version 13.1 released February 3, 2018) available at http://pantherdb.org.

### Effects of a *prp4kb* mutation on GFP expression and development

To test whether a homozygous mutation in *PRP4KB* (At1g13350), the paralog of *PRP4KA*, would affect GFP expression, we crossed the wild-type T line (*T*/*T*;*B*/*B*) with a homozygous *prp4kb* T-DNA insertion mutant (−/−;*b*/*b*) (SALK_035104C). In the *prp4kb* mutant, the T-DNA is inserted in the ninth exon, thus disrupting the PRP4 kinase domain. Self-fertilization of the F_1_ plants generated from this cross (genotype *T*/-;*B*/*b*; the dash indicates hemizygosity for the transgenic *T* locus) yielded a segregating F_2_ population. F_2_ seeds were germinated on solid Murashige and Skoog medium and screened ∼2 weeks later under a fluorescence stereomicroscope for GFP expression, which is observed with a genotype of either *T*/*T* or *T*/- [collectively written hereafter as *T*/(*T*)]. A subset of GFP-positive F_2_ seedlings was transferred to soil for genotyping to identify *T*/(*T*);*b*/*b* plants. To assess the effects of a *prp4kb* mutation on development, the *prp4kb* homozygous mutant was grown on soil next to age-matched *prp4ka* mutants and the wild-type T line. All plants were observed during the entire growth and reproductive phases and into senescence. Characters noted included flowering time, rosette diameter, final height of adult plant, stem/branch number, and silique number.

To investigate the viability of double homozygous mutant plants (*a*/*a*; *b*/*b*), we crossed the homozygous *prp4ka-4* mutant (*T*/*T*;*a*/*a*;*B*/*B*) to a *prp4kb-1* homozygous plant (*A*/*A*;*b*/*b*). Both of these alleles are presumably nulls (Figure S1). Self-fertilization of the F_1_ plants resulting from this cross (genotype *T*/-;*A*/*a*;*B*/*b*) produced a segregating F_2_ population. The F_2_ seeds were germinated on solid Murashige and Skoog medium and prescreened under a fluorescence stereomicroscope for a GFP-weak phenotype [indicating a genotype of *T*/(*T*);*a*/*a* with the *b* allele segregating in the F_2_ population]. Selected GFP-weak F_2_ progeny were transferred to soil for genotyping to identify *T*/(*T*);*a*/*a*;*b*/*b* plants. Primers for detecting *prp4kb-1* are listed in Table S1.

### Data availability

Seeds of the homozygous T line can be acquired from the *Arabidopsis* Biological Resource Center (ABRC), Ohio State University, under the stock number CS69640. Seeds of the *prp4ka* and *sac3a* mutants will be submitted to ABRC upon acceptance of the article and are presently available on request from the Matzke laboratory. RNA and DNA sequencing data are available at the National Center for Biotechnology Information Sequence Read Archive under accession number SRP117313. The mass spectrometry proteomics data have been deposited to the ProteomeXchange Consortium via the PRIDE (Vizcaíno *et al.* 2016) partner repository with the data set identifier PXD008580. Figure S1 shows phenotypes of *sac3a*, *prp4ka*, and *prp4kb* mutants; Figure S2 shows amino acid sequence alignments of PRP4K proteins in selected plant species; Figure S3 shows amino acid alignments of PRP4K proteins in model organisms; Figure S4 shows amino acid alignments of SAC3A proteins in selected plant species; Figure S5 shows amino acid alignments of SAC3A proteins in model organisms; Figure S6 shows a statistical analysis of features of introns affected by differential alternative splicing (DAS) in the *prp4ka* mutant; Figure S7 shows a statistical analysis of features of introns affected by DAS in the *sac3a* mutant; Figure S8 contains an analysis of alternative introns differentially regulated in both *prp4ka* and *sac3a* mutants; Figure S9 contains an analysis of first intron splicing in wild type and the *prp4ka* mutant; Figure S10 is a figure of the spliceosomal cycle and predicted positions of mutated factors identified in the screen; Table S1 shows primers used in this study; Table S2 shows mutants identified so far in the forward genetic screen; Table S3 shows spliceosomal and NineTeen Complex (NTC)-associated genes/proteins changing in expression, alternative splicing, and/or phosphorylation in *prp4ka*; Table S4 shows spliceosomal and NTC-associated genes changing in expression and/or alternative splicing in the *sac3a* mutant; Table S5 shows differentially expressed genes (DEGs) in the *prp4ka* and *sac3a* mutant; Table S6 shows IR events affected in the *prp4ka* and *sac3a* mutants; Table S7 shows ES events affected in the *prp4ka* and *sac3a* mutants; Table S8 shows alternative 5′ and 3′ splice-site events affected in the *prp4ka* or *sac3a* mutants; Table S9 shows exitron splicing events affected in *prp4ka* and *sac3a* mutants; Table S10 lists phosphorylation changes in the *prp4ka* mutant; Table S11 shows a GO analysis for genes affected in the *prp4ka* mutant; Table S12 shows a GO analysis for genes affected the in *sac3a* mutant; Table S13 shows a GO analysis for the shared set of genes affected in the *prp4ka* and *sac3a* mutants; and Table S14 lists flowering genes affected in the *prp4ka* mutant. Supplemental material available at Figshare: https://doi.org/10.25386/genetics.7171694.

## Results

### Alternatively spliced *GFP* reporter gene system

**Figure 1:**
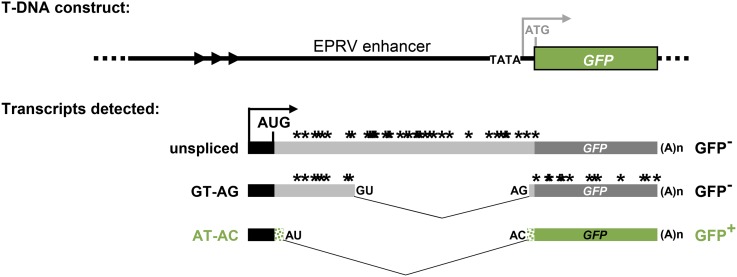
Alternatively-spliced *GFP* reporter gene used in genetic screen. Top: The T-DNA construct introduced into *Arabidopsis* comprises a *GFP* reporter gene under the transcriptional control of a minimal promoter (TATA) and upstream viral (EPRV) enhancer. In the wild-type T line, however, the expected transcription initiation site (gray arrow) is not used. Rather, transcription of *GFP* pre-mRNA initiates at a cryptic upstream promoter (black bar and arrow). Alternative splicing yields three *GFP* splice variants: an unspliced transcript, a transcript resulting from splicing of a canonical GT–AG intron, and a transcript arising from splicing a U2-type intron with noncanonical AT–AC splice sites, which are weakly recognized by the U2 spliceosome compared to canonical GT–AG splice sites (Crotti *et al.* 2007). The unspliced and GT–AG transcripts contain numerous premature termination codons (*). Hence only the AT–AC transcript can be translated into GFP protein. The coding sequence of GFP protein (green bars) uniquely contains a 27 amino acid extension (short stippled green bars) compared to standard GFP (Fu *et al.* 2015; Kanno *et al.* 2016). Arrowheads denote a tandem repeat cluster upstream of the cryptic promoter. AUG designates the major translation initiation codon. The 3′ AT splice site is only 3 nt downstream of the 3′ AG splice site (Kanno *et al.* 2008, 2016, 2017a,b). Figure adapted from figure 1 in Kanno *et al.* (2018).

The alternatively spliced *GFP* reporter gene used in the forward genetic screen to identify splicing factors has been described previously (Sasaki *et al.* 2015; Kanno *et al.* 2016, 2017a,b). Of three major transcripts issuing from the *GFP* reporter gene, only one, which results from splicing a U2-type intron with noncanonical AT–AC splice sites, corresponds to a translatable *GFP* mRNA (Figure 1). The AT–AC intron does not contain the highly conserved 5′ splice-site sequence and branch-point sequence typical of U12 introns (recognized by the minor U12 spliceosome) (Sasaki *et al.* 2015) and hence is most likely spliced by the major U2 spliceosome, which is known to splice AT–AC introns in addition to canonical GT–AG introns (Burge *et al.* 1998; Turunen *et al.* 2013). Mutations in genes encoding splicing proteins can change the ratio of the three transcripts, giving rise to either a GFP-weak (gfw) or Hyper-GFP (hgf) phenotype relative to the intermediate level of GFP observed in the wild-type T line (Sasaki *et al.* 2015; Kanno *et al.* 2016, 2017a,b). So far, we have reported five *hgf* and four *gfw* mutants, all of which are deficient in splicing-related factors predicted to act at various stages of the spliceosomal cycle and small nuclear ribonucleoprotein (snRNP) maturation pathway (Table S2). Here we describe two new mutants in the GFP-weak category: *gfw5* and *gfw6*.

### Recovery of *prp4ka* (*gfw5*) and *sac3a* (*gfw6*) mutants

**Figure 2:**
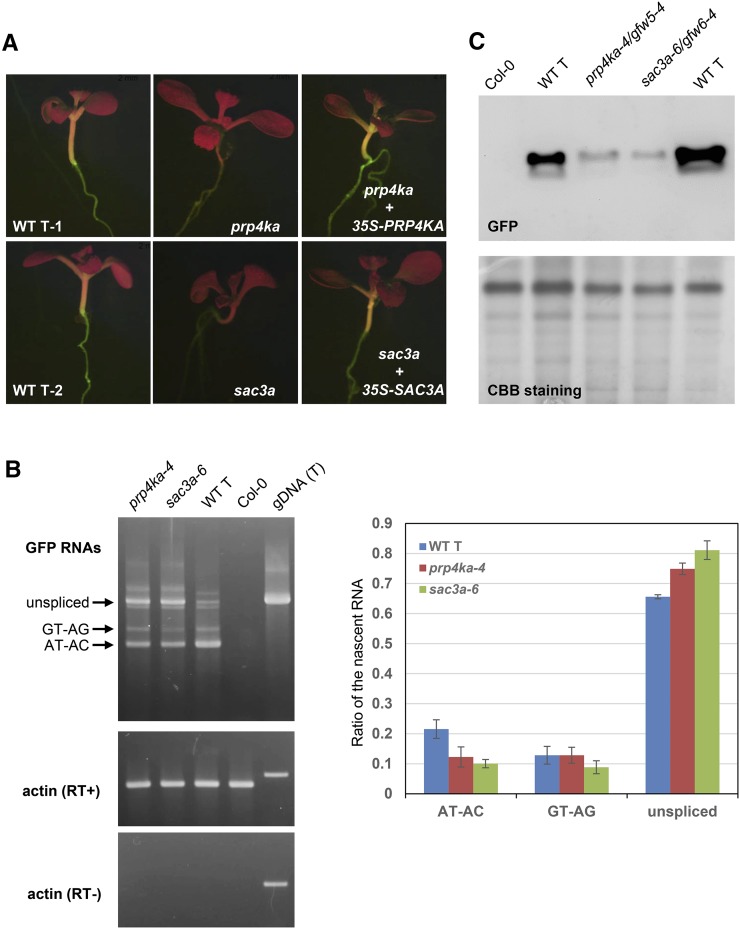
Molecular basis of GFP-weak phenotypes of *prp4ka* and *sac3a* mutants. (A) GFP-weak fluorescence in seedlings of *prp4a* and *sac3a* mutants (*prp4ka-2*/*gfw5-2* and *sac3a-3*/*gfw6-1*). (B) Left: Semiquantitative RT-PCR to detect the three *GFP* splice variants (unspliced, GT–AG transcripts, and AT–AC transcripts) in *prp4ka* and *sac3a* mutants. Wild-type T line and nontransgenic Col-0 represent positive and negative controls, respectively; actin is the constitutively expressed control. Right: Percentages of the three major *GFP* RNA splice variants derived from an analysis of RNA-seq data (Table S5). The average of three biological replicates is shown for each sample. A two-sample *t*-test using the percentages of *GFP* RNA isoforms found a statistically significant difference between the amount of AT–AC and unspliced transcripts between the wild-type T line and the two mutants (*P* < 0.05). The total amount of *GFP* transcripts did not change significantly in *prp4ka* and *sac3a* mutants. (C) Western blotting to detect GFP protein in *prp4ka* and *sac3a* mutants. Total protein isolated from the indicated plant lines was separated by SDS-PAGE, blotted onto a membrane, and probed with a monoclonal antibody to GFP protein (top). The Coomassie brilliant blue-stained gel is shown as a loading control. The prominent ∼56-kDa band is presumed to be the large subunit of ribulose bisphosphate carboxylase. CBB, Coomassie brilliant blue; gDNA (T), genomic DNA of T line; RT−, without reverse transcriptase; RT+, with reverse transcriptase; T, wild-type T line (GFP-intermediate control); WT, wild type.

**Figure 3:**
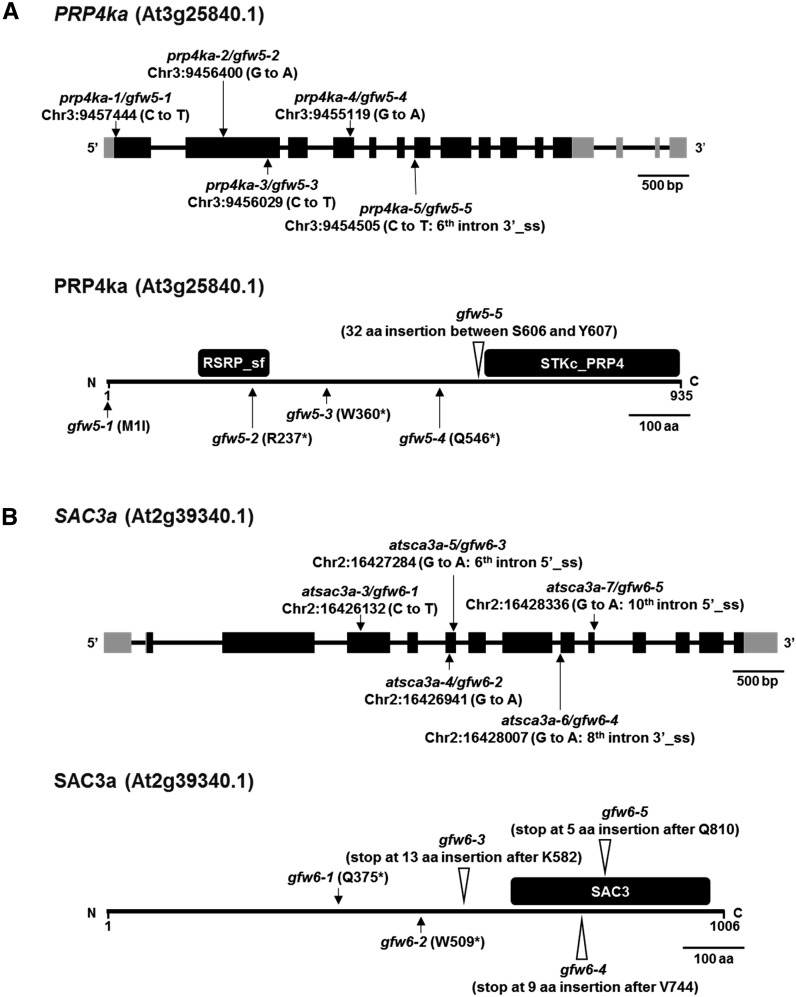
*PRP4KA* and *SAC3A* gene structures, positions of mutations, and protein domains. (A) The pre-mRNA of *PRP4KA* (At3g25840) is alternatively spliced. Two splice variants are annotated in TAIR (http://www.arabidopsis.org/index.jsp) and 14 splice variants are annotated in AtRTD2 (Zhang *et al.* 2017). The reference transcript isoform At3g25840.1 encodes a 935 amino acid protein that contains RS protein (RSRP) superfamily domain and a catalytic domain of the serine/threonine kinase, pre-mRNA processing factor 4 (STKc_PRP4) domain (https://www.ncbi.nlm.nih.gov/). We identified the following *prp4ka* alleles in our screen: *prp4ka-1* (M1I), *prp4ka-2* (R237*), *prp4ka-3* (W360*), *prp4ka-4* (Q546*), and *prp4ka-5* (splice-site acceptor, sixth intron) (Figure S2). All of these alleles, which encode defective mRNAs or, in one case, abolishes initiation of translation at the normal ATG start codon (the next methionine codon is 300 bp downstream), are likely to be nulls. (B) *SAC3A* (At2g39340) encodes a 1066 amino acid protein containing a conserved SAC3/GANP/THP3 domain (http://www.arabidopsis.org/). In budding yeast, this domain in the SAC3 protein integrates interactions between other proteins in the TREX complex that couples transcription and mRNA export (https://www.ebi.ac.uk/interpro/). We identified the following *sac3a* alleles: *sac3a-3* (Q375*), *sac3a-4* (W509*), *sac3a-5* (splice-site donor, sixth intron), *sac3a-6* (splice-site acceptor, eighth intron), and *sac3a-7* (splice-site donor, 10th intron). The *sac3a* alleles all encode defective mRNAs and are likely to be nulls. Chr3, chromosome 3; 3′_ss, alternative 3′ splice-site acceptor; 5′_ss, alternative 5′ splice-site donor.

The *gfw5* and *gfw6* mutants were identified by their GFP-weak phenotypes in a population of M_2_ seedlings (Figure 2A). NGM (James *et al.* 2013) using pooled DNA isolated from at least 50 GFP-weak BC_1_F_2_ seedlings of the *gfw5* and *gfw6* mutants revealed homozygous recessive mutations in genes encoding PRP4KA and SAC3A, respectively. Subsequent Sanger sequencing of *PRP4KA* and *SAC3A* genes in additional unnamed *gfw* mutants identified a total of five *prp4ka* alleles and five *sac3a* alleles. The *prp4ka* alleles are the first to be isolated and hence are named *prp4ka-1* to *prp4ka-5* (Figure 3A). In view of two T-DNA insertion alleles previously published for *sac3a* (Lu *et al.* 2010), the new *sac3a* alleles are designated *sac3a-3* to *sac3a-7* (Figure 3B). Complementation of the *prp4ka-4* and *sac3a-6* mutants with the respective wild-type coding sequences resulted in restoration of intermediate, wild-type levels of GFP fluorescence (Figure 2A), thus confirming that the *prp4ka* and *sac3a* mutations were responsible for the GFP-weak phenotypes of the respective mutants.

The positions of the *prp4ka* and *sac3a* mutations are shown in Figure 3. *PRP4KA*, which encodes a protein 935 amino acids in length, has two paralogs in *Arabidopsis*: *PRP4KB* (At1g13350; 834 amino acids) and At3g53640, which is an intronless, unexpressed pseudogene. Both *PRP4KA* and *PRP4KB* are ubiquitously expressed, with *PRP4KA* having a higher expression level than *PRP4KB* (http://bar.utoronto.ca/efp/cgi-bin/efpWeb.cgi). *SAC3A* has two expressed paralogs in *Arabidopsis*, *SAC3B* (At3g06290) and *SAC3C* (At3g54380), which are more closely related to each other than to *SAC3A* (Lu *et al.* 2010). Coexpression analysis using both the ATTED database version 9.2 (http://atted.jp/; CoExSearch function) and the Expression Angler tool (http://bar.utoronto.ca/; AtGenExpress Plus – Extended Tissue Compendium) indicated that *PRP4KA* and *SAC3A* are highly coexpressed in *Arabidopsis*. Amino acid sequence alignments of PRP4KA and SAC3A orthologs in selected plant species and model organisms are shown in Figures S2–S5, respectively.

### Characterization of *prp4ka* and *sac3a* mutants

Semiquantitative RT-PCR was used to investigate the splicing pattern of *GFP* pre-mRNA in *prp4ka-4* and *sac3a-6* mutants. Relative to the wild-type T line, the mutants accumulated reduced amounts of the translatable AT–AC *GFP* transcript and increased levels of the unspliced, untranslatable transcript (Figure 2B). Western blot analysis demonstrated decreased levels of GFP protein in *prp4ka* and *sac3a* mutants (Figure 2C). These results are consistent with the GFP-weak phenotype of M_2_ seedlings.

**Figure 4:**
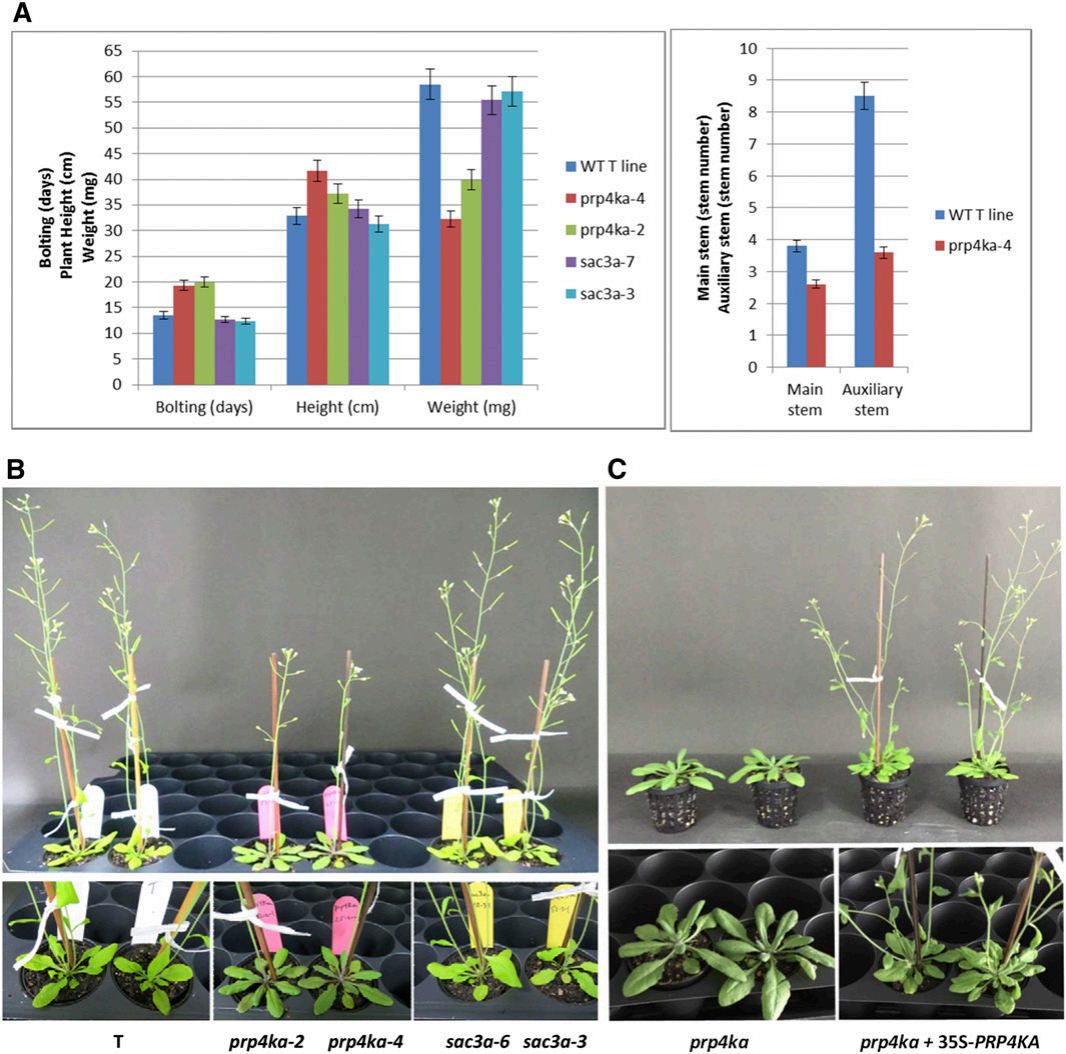
Phenotypic analysis of *prp4ka* and *sac3a* mutants. (A and B) The *sac3a* mutants appear largely normal by the measured criteria: transition to flowering (bolting), final height of adult plant, seed weight, and branch (stem) number. The *prp4ka* mutants feature delayed flowering, lowered seed set, reduced branching, a tall final stature, and somewhat flat, darker green rosettes [numerical values in Figure S1A (*prp4ka* and *sac3a* experiment) and Figure S1B (*prp4kb* experiment)]. (B) Tall stature is not visible in the photograph, which shows age-matched, wild-type (WT) and mutant plants, but is apparent in fully grown *prp4ka* plants (Figure S1A). (C) Complementation of the *prp4ka* mutants with a 35Spro-*PRP4KA* transgene restores a normal phenotype. Particularly visible in the age-matched samples shown here is the late transition to flowering and somewhat flat, darker green rosettes in the *prp4ka* mutant (left) compared to the complemented lines (right), which also have normal branching patterns (Figure S1B).

The morphological phenotypes of the *prp4ka* and *sac3a* mutants were examined during vegetative and reproductive phases. Whereas the *sac3a* mutants were largely indistinguishable from wild-type plants, the *prp4ka* mutants displayed a pleiotropic phenotype typified by somewhat flat, darker green rosettes, late flowering, tall final stature, lowered seed set, and reduced branching (Figure 4, A and B, and Figure S1, A and B). The lowered seed set in *prp4ka* mutants reflected both fewer seeds per silique and fewer siliques per plant (Figure S1B). The aberrant traits of *prp4ka* mutants returned to more wild-type levels in complemented plants (Figure 4C and Figure S1, A and B), indicating that the *prp4ka* mutations are indeed largely responsible for the abnormal phenotype of the corresponding mutants.

### RNA-seq analysis

To analyze the genome-wide effects of homozygous *prp4ka* and *sac3a* mutations on differential gene expression and alternative splicing, we carried out RNA-seq using total RNA isolated from 2-week-old seedlings of the homozygous *prp4ka-4* and *sac3a-6* mutants (BC_1_F_3_ generation) and the wild-type T line. All samples were run in biological triplicate.

The RNA-seq data confirmed the findings obtained from semiquantitative RT-PCR, showing reduced splicing efficiency of *GFP* pre-mRNA. The amount of AT–AC transcript decreased significantly in *prp4ka-4* and *sac3a-6* mutants compared to the wild type. By contrast, the level of unspliced, untranslatable transcript increased significantly in *prp4ka-4* and *sac3a-6* mutants relative to the wild type (Figure 2B).

The findings from a genome-wide analysis of DEGs and DAS are summarized in Table 1. A number of splicing-related factors were identified in this analysis and are compiled separately for *prp4ka* (Table S3) and *sac3a* (Table S4). DEGs numbered 1571 in the *prp4ka* and 3046 in the *sac3a* mutant (Table 1). Of these, around a quarter (407) was shared between the *prp4ka* and *sac3a* mutants, but the direction of change (up or down) was not always the same in both mutants (Table 1 and Table S5). Upregulated genes in the *prp4ka* mutant included *SAC3A* and the putative U1 snRNP component *PRP39A* (Table S5), which was identified previously in the same genetic screen that retrieved the *prp4ka* and *sac3a* mutants (Table S2).

**Table 1:** Summary of DEGs and DAS events in the *prp4ka* and *sac3a* mutants

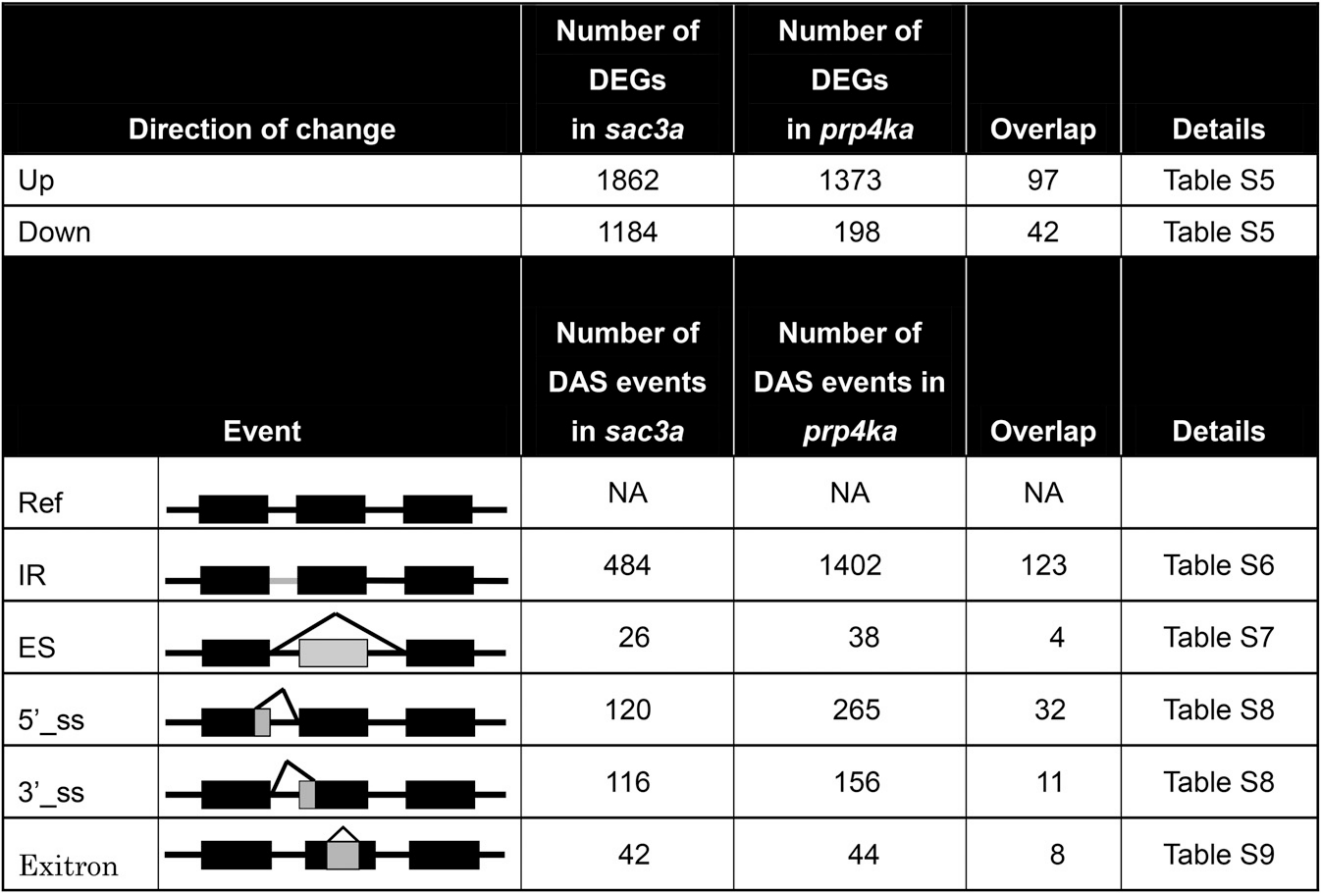					

Ref, reference; 5′_ss, alternative 5′ splice-site donor; 3′_ss, alternative 3′ splice-site acceptor.

a Number of DEGs in the *sac3a* and *prp4ka* mutants using an FDR <0.05.

b The major alternative splicing events are illustrated to the right. Regions included or excluded due to alternative splicing are shown in gray. The numbers of DAS events observed in each mutant are indicated in the middle columns. Overlap columns show the numbers of DEGs and DAS events shared between the *prp4ka* and *sac3a* mutants.

**Figure 5:**
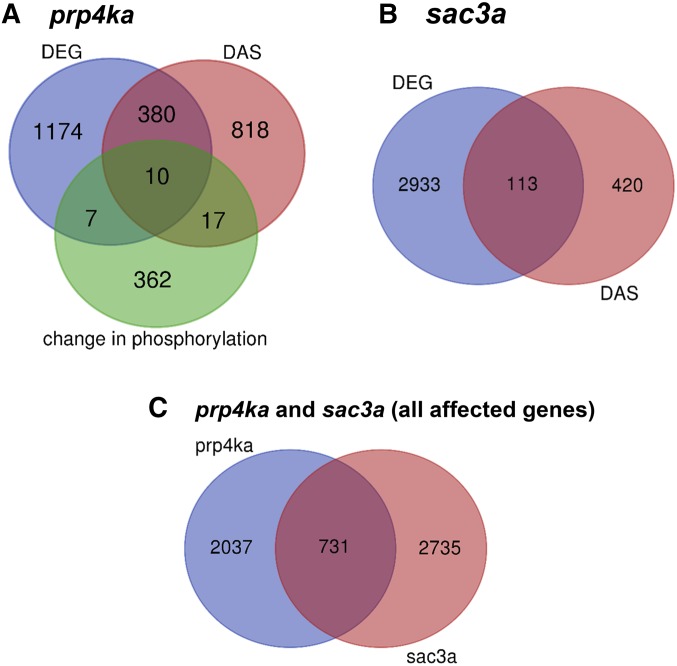
Venn diagrams showing distribution of genes affected in the *prp4ka* and *sac3a* mutants. (A) Venn diagram for genes affected in the *prp4ka* mutant (DEGs, DAS genes, genes encoding proteins with changes in phosphorylation). (B) Venn diagram for genes affected in the *sac3a* mutant (DEGs and DAS genes). (C) Venn diagram for all genes affected in the *prp4ka* (DEGs, DAS genes, changes in phosphorylation) and *sac3a* (DEGs and DAS genes) mutants.

DAS was detected for 1225 and 533 genes in the *prp4ka* and *sac3a* mutants, respectively (Tables S6–S9). The numbers of overlapping genes affected by both DEG and DAS events in the mutants are shown in Figure 5. Whereas about a quarter (390) of *prp4ka* DEGs were also differentially spliced, only ∼3.7% (113) of *sac3a* DEGs displayed changes in alternative splicing (Figure 5). A total of 206 genes showed DAS in both mutants (Tables S6–S9).

In total, 1905 and 788 instances of DAS were detected in the *prp4ka* and *sac3a* mutants, respectively (Tables S6–S9). IR represented the most common DAS event, comprising 1402 cases of differential IR in the *prp4ka* mutant and 484 in the *sac3a* mutant (Table 1 and Table S6). Of these, 123 IR events were shared and the direction of the change for ∼71.5% of them (88 IRs) was the same in both mutants. The vast majority (95.8%) of IRs affected in the *prp4ka* mutant and ∼64% of IRs in the *sac3a* mutant showed higher retention in comparison to controls (Table S6). ES was represented by relatively few events: 38 in the *prp4ka* and 26 in *sac3a*, 4 of which were shared by both mutants although the direction of change was not always the same (Table 1 and Table S7). Several hundred events involving alternative 5′ and 3′ splice-site selection were detected in both mutants (Table 1 and Table S8). A total of 32 alternative 5′ splice-site selection and 11 alternative 3′ splice-site selection events were shared between *prp4ka* and *sac3a* mutants, and the direction of the change was the same for 24 (75%) and 7 (∼63%) of them, respectively (Table S8). Exitrons were represented by 44 and 42 cases in the *prp4ka* and *sac3a* mutants, respectively (Table 1 and Table S9). Eight exitrons overlapped in the two mutants, and all but one, At1g77080, was regulated in the same direction (Table S9). Of all DAS events, only 13 in the *prp4ka* and 3 in the *sac3a* mutants involved a U12 intron; the remainder (1892 in *prp4ka* and 785 in *sac3a*) entailed U2 introns.

### Analysis of alternative introns differentially regulated in *prp4ka* and *sac3a* mutants

We analyzed the guanine-cytosine (GC) content, length, and splice-site strength of introns affected in the *prp4ka* (Figure S6) and *sac3a* (Figure S7) mutants and in two subgroups of alternative splicing events regulated in both mutants: the “shared”-subgroup of 178 events regulated in both mutants, and the “same”-subgroup of 128 events regulated in the same direction (Figure S8). This analysis (Figures S6 and S7) revealed that, in both the *prp4ka* and *sac3a* mutants, the more retained-introns have significantly lower 5′ splice-site scores (*i.e.*, weaker 5′ splice sites; two-sample *t*-test, *P* < 0.001), whereas the 3′ splice-site strength of more-retained introns in *prp4ka* is slightly, but significantly, higher (two-sample *t*-test, *P* < 0.001). Additionally, the more-retained introns of both mutants exhibit a slight, but significant, increase in GC content and the more-retained introns in *prp4ka* are significantly longer (two-sample *t*-test, *P* < 0.001). The alternatively regulated 5′ and 3′ splice-site events in both mutants exhibited no significant difference in splice-site strength. However, the selection of the lesser-used alternative 5′ or 3′ splice site results in significantly longer introns (two-sample *t*-test, *P* < 0.001).

The introns regulated in both the *prp4ka* and *sac3a* mutants (shared- and same-subgroups) are significantly (Wilcoxon test, *P* < 0.05) longer than the introns regulated in either one of the separate mutants (Figure S8A). The GC content of the affected introns in *prp4ka* is lower than those regulated in the *sac3a* mutant (Wilcoxon test, *P* < 0.05) and in both mutants (Figure S8B; Wilcoxon test, *P* < 0.05). The regulated introns in *sac3a* have on average a slightly higher 5′ splice-site score (*i.e.*, stronger 5′ splice sites) (Figure S8C; Wilcoxon test, *P* < 0.001), whereas their 3′ splice-site score is slightly lower than the regulated introns in *prp4ka* (Wilcoxon test, *P* < 0.001) and the regulated introns in both mutants (Figure S8D; Wilcoxon test, *P* = 0.04).

### Analysis of first intron splicing in the *prp4ka* mutant

A previous study in fission yeast found that Prp4 kinase is required for recognition and splicing of a subset of first introns that have weak 5′ splice sites and branch-point sequences (Eckert *et al.* 2016). We evaluated whether the same trend might be observed with the significantly differentially retained introns identified in the *prp4ka* mutant (Figure S9). The regulated introns were split up into those affecting the first introns of a gene and all other (the remaining) introns. There was no significant difference in the 5′ splice-site strength in the first and the remaining introns, whereas the 3′ splice-site score of the first introns was slightly, but significantly, higher (Wilcoxon test, *P* = 0.014). The first introns in the *prp4ka* mutant exhibit significantly higher retention rates compared to the remaining introns (Wilcoxon test, *P* < 0.001), whereas this behavior was not observed for the first and remaining retained introns in wild type. Out of all differentially retained introns in the *prp4ka* mutant, 41.7% (585 out of 1402) comprised first introns. Evaluation of all IR events annotated in the AtRTD2 transcriptome revealed that 27.2% describe the retention of a first intron, which is substantially lower (Chi-square goodness-of-fit test, *P* < 2.2*e*^−16^) than the 41.7% observed in the *prp4ka* mutant.

### Peptide phosphorylation changes detected in the *prp4ka* mutant

To identify potential substrates of PRP4KA, we used the iTRAQ method (Lan *et al.* 2011) to perform a quantitative phosphoproteomic analysis on total protein isolated from 2-week-old seedlings of the *prp4ka-4* mutant and from the wild-type T line. The experiments were performed using three independent biological replicates. Search of the mass spectrometry data against the AtRTD2 translation (Zhang *et al.* 2017) database identified 1059 peptides in proteins encoded by 396 genes. Peptides showing statistically significant changes in phosphorylation in at least two of the three experiments are listed in Table S10. The numbers of overlapping genes/proteins affected by DEG, DAS, or phosphorylation changes in the *prp4ka* mutant are shown in Figure 5A. Twenty splicing-related factors, including five SR proteins (At-SR30, At-RS41, At-RS40, At-SCL33, and At-SCL30A), were identified in the iTRAQ analysis as were a number of other RNA-binding proteins (Table 2 and Table S10). Two splicing factors, AtGRP7 and FLK, showed changes in both phosphorylation levels and alternative splicing in the *prp4ka* mutant (Table S3).

**Table 2:** Phosphorylation changes in selected splicing factors and RNA-binding proteins in the *prp4ka-4* mutant

Peptide sequence*^a^*	Identifier*^b^*	Name	Function	Reference
Lose phosphorylation				
[-].ME**ST**E**S**YAAG**S**PEELAK.[R]	AT5G04430.ID11	BTR1L	NOVA-like RNA-binding protein	de la Fuente van Bentem *et al.* (2006)
[-].ME**ST**E**S**YAAG**S**PEELAKR.[S]	AT5G04430.JS1			
[-].ME**ST**E**SY**AAG**S**PEELAKR**S**PEPHD**SS**EAD**S**AEKP**T**HIR.[F]	AT5G04430.JS4			
	AT5G04430.P1			
	**AT5G04430.P2**			
[K].EGGGYSFFP**S**PSANGAQGALTYQ.[-]	**AT3G21215.P1**	RNA-binding (RRM/RBD/RNP motifs) family protein	A putative RNA splicing protein similar to mec-8	AceView (https://www.ncbi.nlm.nih.gov/IEB/Research/Acembly/)
[K].RKEGGGYSFFP**S**PSANGAQGALTYQ.[-]				
[R].AA**S**PQIR**ST**PEID**SS**Q**Y**L**T**ELLAEHQK.[L]	AT2G38610.P2	RNA-binding KH domain-containing protein	Similar to human QKI proteins; regulation of pre-mRNA splicing, export of target RNAs from the nucleus, translation of proteins, and RNA stability	AceView (https://www.ncbi.nlm.nih.gov/IEB/Research/Acembly/)
		Quaking-like 3		
[R].SVPSSPGPNWLN**S**PGSSSGLIAK.[R]	**AT5G56140.P1**	RNA-binding KH domain-containing protein		
[R].SVPSSPGPNWLN**S**PGSSSGLIAKR.[T]		Quaking-like 2		
[K].IFVGGISY**S**TDEFGLR.[E]	AT1G74230.ID1	GR-RBP5	Glycine-rich RNA-binding protein; hnRNP family	Table S1 in Koncz *et al.* (2012)
	AT1G74230.ID4			
[R].SGGGGGYSGGGG**S**YGGGGGR.[R]	**AT2G21660.P1**	CCR2/ATGRP7	Glycine-rich RNA-binding protein	Table S1 in Koncz *et al.* (2012)
[R].SGGGGGYSGGGG**S**YGGGGGRR.[E]				
[K].VVVAYGG**T**PIHQQLR.[E]	AT3G58510.3	DEA(D/H)-box RNA helicase family protein	B complex associated	Table S1 in Koncz *et al.* (2012)
[R].FSP**S**VDR.[Y]	AT1G09140.CR4	At-SR30	SR protein, SR subfamily	Barta *et al.* (2010); Table S1 in Koncz *et al.* (2012)
[R].FSP**S**VDRYSSSYSASR.[A]	AT1G09140.ID154			
	AT1G09140.ID155			
	AT1G09140.ID157			
	AT1G09140.ID85			
	**AT1G09140.P1**			
	AT1G09140.P2			
	AT1G09140.P3			
[K].DDDSRGNGY**S**PER.[R]	AT5G52040.ID8	At-RS41	SR proteins, plant-specific RS subfamily	de la Fuente van Bentem *et al.* (2006); Barta *et al.* (2010); Table S1 in Koncz *et al.* (2012)
[R].GNGY**S**PER.[R]	AT5G52040.ID14			
[R].GNGY**S**PERR.[R]				
[RK].ER**TS**PD**Y**GR.[GR]				
[RK].ER**TS**PD**Y**GR.[GR]	AT4G25500.4	At-RS40		
[M].**S**GGLDMSLDDIIK.[S]	AT5G02530.2	ALY2	TREX complex	Table S1 in Koncz *et al.* (2012)
	**AT5G02530.P1**			
[R].N**T**FDENVD**S**NNNL**S**P**S**A**S**QGIGAP**S**P**YSY**AAVLG**SS**L**S**R.[N]	**AT2G29200.P1**	Pumilio 1 (PUM1)	PUF proteins regulate both mRNA stability and translation through sequence-specific binding to 3′ UTRs of target mRNA transcripts	
[R].**S**GSAPPTVDGSVSAAGGLFSGGGGAPFLEFGGVNK.[G]				
[R].**S**GSAPPTVDGSVSAAGGLFSGGGGAPFLEFGGVNKGNGFGGDDEEFR.[K]				
[K].NNL**S**PSASQGIGAPSPYSYAAVLGSSLSR.[N]	AT2G29190.2	Pumilio 2 (PUM2)		
[R].**S**GSAPPTVDGSVSAAGGLFSGGGGAPFLEFGGGNK.[G]				
[R].**S**GSAPPTVDGSVSAAGGLFSGGGGAPFLEFGGGNKGNGFGGDDEEFR.[K]				
[K].SIADMIQRPH**S**AGNRPIAQDIHAISSDTSSEHAR.[R]	AT3G20250.c2	Pumilio 5 (PUM5)		
	AT3G20250.c3			
	AT3G20250.c4			
	AT3G20250.ID3			
	AT3G20250.ID4			
	AT3G20250.JC6			
[R].D**S**PTSQPVPIVALATR.[L]	AT1G49760.2	Poly(A) binding protein 8 (PAB8)	mRNA binding protein	Table S1 in Koncz *et al.* (2012)
[R].DVN**T**MPGP**T**QNML**S**VP**Y**DV**SS**GGGVHHRD**S**P**TS**QPVPIVALA**T**R.[L]				
[R].EL**S**P**T**GLD**SS**PR.[D]	AT3G51950.ID3	Zinc finger (CCCH-type) family protein/RNA recognition motif (RRM)-containing protein	Ribonuclease activity	Addepalli and Hunt (2008)
[R].EL**S**P**T**GLD**SS**PRDVLGGR.[G]				
[R].**S**GSCVLDGLGYGGDSDLGFGGVPCSYFAR.[G]				
[K].FRIP**S**PGDVYNR.[T]	AT5G60170.1	RNA-binding (RRM/RBD/RNP motifs) family protein	Similar to human CCR4-NOT transcription complex, subunit 4	AceView (https://www.ncbi.nlm.nih.gov/IEB/Research/Acembly/)
[R].NL**S**PSLNDPYGFSSR.[L]	AT5G60170.ID35			
Gain phosphorylation				
[K].GNPLLN**T**PTSFSVK.[R]	**AT3G13200.P1**	Splicing factor Cwf15/Cwc15	NTC-associated	Table S1 in Koncz *et al.* (2012)
[K].KSLNRSPPS**Y**GSHPR.[G]	**AT3G13224.P2**	RNA-binding glycine-rich protein D3 (RBGD3)	Glycine-rich RNA-binding protein; hnRNP family	
[K].SLNRSPPS**Y**GSHPR.[G]	AT3G13224.P3			
[K].NG**S**VSGTELVEDDHER.[A]	AT5G16260.c1	Early flowering 9 (ELF9)	17S U2 snRNP	Table S1 in Koncz *et al.* (2012)
[R].LKNG**S**VSGTELVEDDHER.[A]	**AT5G16260.P1**			
[K].VEDEEGIPEHLE**S**LQK.[S]	AT3G04610.ID6	Flowering locus KH domain (FLK)	hnRNP E1/E2	Table S1 in Koncz *et al.* (2012)
	AT3G04610.JC12			
	AT3G04610.JC3			
	AT3G04610.P2			
	AT3G04610.P4			
	AT3G04610.s3			
[R].EEG**S**PM**S**G**S**I**S**P**Y**N**S**LGMK.[R]	**AT4G26480.P1**	RNA-binding KH domain-containing protein	Similar to human QKI proteins; regulation of pre-RNA splicing, export of target RNAs from the nucleus, translation of proteins, and RNA stability	AceView (https://www.ncbi.nlm.nih.gov/IEB/Research/Acembly/)
		Quaking-like 1		
[R].S**T**PEIDSSQYLTELLAEHQK.[L]	AT2G38610.P2	RNA-binding KH domain-containing protein		
		Quaking-like 3		
[R].EEGSPMSGSV**S**PYNSLGMK.[R]	**AT5G56140.P1**	RNA-binding KH domain-containing protein		
		Quaking-like X3		
[R].SY**T**P**S**PPR.[G]	AT1G55310.2	At-SCL33	SR proteins, plant-specific SCL subfamily	de la Fuente van Bentem *et al.* (2006); Barta *et al.* (2010); Table S1 in Koncz *et al.* (2012)
	**AT1G55310.3**			
	AT1G55310.c2			
	AT1G55310.c3			
	AT1G55310.CR7			
	AT1G55310.ID1			
	AT1G55310.ID3			
	AT1G55310.P1			
	AT1G55310.P3			
[R].SY**T**P**S**PPRGYGR.[R]	AT3G13570.c1	At-SCL30A		
	AT3G13570.CR2			
	**AT3G13570.P1**			
	AT3G13570.SR1			
[R].MLQSGMPLDDRPEGQR**S**P**S**PEPVYDNMGIR.[I]	**AT5G51300.1**	atSF1/BBP splicing factor 1	Splice site selection	de la Fuente van Bentem *et al.* (2006); Table S1 in Koncz *et al.* (2012)
[R].**S**P**S**PEPVYDNMGIR.[I]				
[R].**S**G**S**APP**T**VDG**S**V**S**AAGGLF**S**GGGGAPFLEFGGVNK.[G]	**AT2G29200.P1**	pumilio 1 (PUM1)	PUF proteins regulate both mRNA stability and translation through sequence-specific binding to 3′ UTRs of target mRNA transcripts	
[R].**S**G**S**APP**T**VDG**S**V**S**AAGGLF**S**GGGGAPFLEFGGGNK.[G]	AT2G29190.2	Pumilio 2 (PUM2)		
[R].DAALGSQLSRPA**S**CNTFR.[D]	AT3G10360.JC2	Pumilio 4 (PUM4)		
[R].GNF**S**PGSSPSGMDSR.[D]	**AT3G21100.2**	RNA-binding (RRM/RBD/RNP motifs) family protein		
	AT3G21100.ID3			
	AT3G21100.ID8			
[K].DSNVTPDDDVSGMR**S**PSAFFK.[H]	**AT3G13300.P1**	Varicose (VCS)	Involved in mRNA decapping	
[K].VFCSQVSNL**S**TEMAR.[D]	AT3G13300.P2			
[R].DCYP**S**TEGTFIPGESK.[A]	AT3G13300.P3			
[K].**SSS**AAD**SY**VG**S**LI**S**L**TS**K.[S]	AT1G26110.2	Decapping 5 (DCP5)	mRNA decapping	
	AT1G26110.ID1			
	AT1G26110.ID2			
	AT1G26110.ID5			
	**AT1G26110.P1**			
[K].**S**PVATTQQLPK.[V]	AT1G79280.1	Nuclear pore anchor (NUA)	mRNA export	Table S1 in Koncz *et al.* (2012)
[R].VPSSTPLIK**S**PVATTQQLPK.[V]	**AT1G79280.2**			
[R].VP**S**STPLIK.[S]	AT1G79280.3			
[K].VVM**T**PDTPSK.[G]	AT3G62800.2	Double-stranded-RNA-binding protein 4 (DRB4)	A nuclear dsRNA-binding protein DRB4 that interacts specifically with DCL4	
	AT3G62800.P3			
[R].DGPGPLH**S**PAVSK.[S]	AT5G57870.2	Eukaryotic translation initiation factor isoform 4G1 (eIFiso4G1)	RNA metabolic process	
[R].RDGPGPLH**S**PAVSK.[S]	**AT5G57870.P1**			

NOVA-1, a mammalian, neuron-specific regulator of alternative splicing containing three K homology domains; mec-8, a *Caenorhabditis elegans* protein that regulates alternative splicing of unc-52; KH, K homology.

a Serines (S), threonines (T), and tyrosines (Y) in bold font and which are underlined indicate the phosphorylated residues detected by iTRAQ. The peptides listed showed statistically significant changes in phosphorylation in at least two out of three separate iTRAQ experiments (Table S10). The amino acids before and after the tryptic peptide in the protein sequence are annotated by brackets and separated by dots.

b Gene models (identifiers) are according to the AtRTD2 transcriptome annotation (Zhang *et al.* 2017). Reference gene models are shown in bold font. For a fuller list of RNA metabolism-related proteins identified in the iTRAQ analysis see Tables S2 and S10 (see “Keyword RNA”).

### GO analyses of genes affected in *prp4ka* and *sac3a* mutants

The overrepresentation test of GO terms for all 2768 genes whose expression is affected at different levels (DE, DAS, and changes in phosphorylation) in the *prp4ka* mutant (Figure 5A) shows enrichment of RNA-processing and splicing-related terms (Table S11). Although these terms were not overrepresented in the *prp4ka* DEGs (1571 genes), they were among the most highly enriched GO terms for DAS genes (1225). Similarly, for the set of proteins with phosphorylation changes (396 genes), RNA-processing and splicing-related terms were also overrepresented (Table S11).

For the 3466 genes affected (DE and DAS) in the *sac3a* mutant (Figure 5B), the overrepresented terms included “RNA binding protein” and “spliceosomal complex” (Table S12). For the 3046 DEGs in *sac3a*, similar terms were overrepresented with the exception of spliceosomal complex. By contrast, significant enrichment of splicing-related terms was observed for the 533 DAS genes (Table S12).

For genes/proteins affected by DEG, DAS, or phosphorylation in *prp4ka* (2768) and DEG or DAS in *sac3a* (3466), 731 were shared (Figure 5C). GO analysis showed enrichment of the terms “nuclear speckle” and spliceosomal complex (Table S13).

### Tests of a *prp4kb* mutation on *GFP* expression and plant phenotype

To investigate whether a homozygous mutation in PRP4KBwould confer a GFP-weak phenotype similar to *prp4ka* mutations, we performed the breeding scheme described in the *Materials and Methods*. Of 23 GFP-intermediate F_2_ plants descending from a cross between a homozygous *prp4kb-1* mutant (−/−; *b/b*) and the wild-type T line (*T*/*T*; *B*/*B*), four (17.4%, expected percentage 25%) were found to be homozygous for the *prp4kb-1* mutation. The finding of homozygous *b*/*b* F_2_ plants with intermediate, wild-type levels of GFP fluorescence demonstrates that a *prp4ka* mutation does not weaken *GFP* expression. Homozygous *prp4kb-1* plants appear normal, in contrast to the aberrant phenotype of *prp4ka* mutants (Figure S1, B and C).

To assess the viability of plants homozygous for both the *prp4ka-4* and *prp4kb-1* mutations, we performed the breeding strategy described in the *Materials and Methods* section. F_3_ progeny of a *T*/(*T*);*a*/*a*;*B*/*b* plant were prescreened for a GFP-weak phenotype [indicating homozygosity of the *prp4ka-4* allele or *T*/(*T*);*a*/*a*]. We genotyped 54 GFP-weak F_3_ progeny for the *prp4kb-1* allele and found 5 that were heterozygous for the *prp4kb-1* mutation [*T*(*T*);*a*/*a*;*B*/*b*]. However, no doubly homozygous F_3_ progeny [*T*/(*T*);*a*/*a*;*b*/*b*] were identified. If the double homozygous mutant is viable, the expected number of *T*/(*T*);*a*/*a*;*b*/*b* F_3_ progeny in a population of 54 plants would be 13–14 (25%). These results suggest that the double homozygous mutant is not capable of survival. However, the number of *B*/*b* heterozygotes obtained in the F_3_ population (5 out of 54 or 9.25%) was also lower than expected (27 out of 54 or 50%), which may indicate that the *b* allele is not transmitted well in the *a*/*a* mutant background.

## Discussion

In a forward genetic screen for mutants showing modified splicing of an alternatively spliced *GFP* reporter gene in *Arabidopsis*, we recovered loss-of-function mutations in the genes encoding the dual-specificity protein kinase PRP4KA and the putative mRNA nuclear export factor SAC3A. Both the *prp4ka* and *sac3a* mutants were identified by their GFP-weak phenotypes, which are due—at least in part—to diminished splicing efficiency of *GFP* pre-mRNA. PRP4KA and SAC3A have not been identified in any prior forward genetic screen in *Arabidopsis* or studied previously for their roles in pre-mRNA splicing in plants. It is unclear why this particular screen repeatedly retrieved mutants defective in these two genes, but the findings clearly demonstrate the contributions of PRP4KA and SAC3A to *GFP* pre-mRNA splicing and to *GFP* expression.

### PRP4KA

PRP4K-related proteins are present in most eukaryotes with the prominent exception of the fungal group Hemiascomycetes, which contains budding yeast. Prp4 kinase is an essential gene in fission yeast (Alahari *et al.* 1993; Lützelberger and Käufer 2012) and in metazoans (Dellaire *et al.* 2002). By contrast, our study indicates that PRP4KA is not essential in *Arabidopsis*. Prp4 kinase is also not necessary for growth in the wheat scab fungus *Fusarium graminaerum* but it is needed for efficient splicing (Gao *et al.* 2016). Although the *prp4ka* alleles we identified are most likely genetic nulls, the respective mutants are viable and fertile. Nevertheless, they show an obvious pleiotropic phenotype, the molecular basis of which remains to be established. The DEG, DAG, and protein phosphorylation lists may suggest candidate genes for follow-up studies; for example, these lists contain a number of flowering-related genes, which may contribute to the late-flowering phenotype (Table S14).

The failure of a *prp4kb* mutation to visibly affect either *GFP* expression or plant morphology and development rules out extensive functional redundancy of *PRP4KA* and *PRP4KB*. This conclusion is supported by the fact that we retrieved five independent mutant alleles of *prp4ka* in our screen but not a single mutant allele of *prp4kb*. The inability to recover *prp4ka prp4kb* double mutants indicates that at least one wild-type copy of a *PRP4K* gene is essential for plant viability. However, this possibility needs to be examined more thoroughly in the future by reassessing the apparent weak inheritance of the *prp4kb-1* allele in the homozygous *prp4ka-4* mutant, which itself displays reduced fertility as evidenced by a lowered seed set.

Genetic studies in fission yeast (Bottner *et al.* 2005) and *F. graminaerum* (Gao *et al.* 2016) as well as biochemical analyses in human cells (Schneider *et al.* 2010; Boesler *et al.* 2016) established that Prp4 kinase transiently associates with the spliceosome as a component of the precatalytic B complex and facilitates the transition to the catalytically active B* (or B^act^) complex (Schneider *et al.* 2010). Determining whether PRP4KA has a similar role in splicing in *Arabidopsis* will require the development of methods for isolating the cognate spliceosomal complexes from plant cells. Although detailed biochemical analyses of plant spliceosomes await further technical advances, a PRP4KA–GFP fusion protein in *Arabidopsis* was localized to nuclear speckles, which are enriched in splicing factors, thus further substantiating a role for PRP4KA in splicing (Koroleva *et al.* 2005).

Both PRP4KA and another splicing factor identified previously in this screen, SMU1 (Kanno *et al.* 2017a), are placed in the category “recruited prior to B^act^” (the spliceosomal complex preceding catalytic B*) in a compilation of known and predicted splicing factors in *Arabidopsis* (table S1 in Koncz *et al.* 2012). In human cells, Smu1, like Prp4k, is most abundant in the precatalytic B complex (Wahl and Lührmann 2015) and has been proposed to act by recognizing splicesomal targets for ubiquitination (Higa *et al.* 2006). Based on these findings from human cells, one can speculate that PRP4KA and SMU1 in *Arabidopsis* are likewise components of the precatalytic B complex and are involved in triggering different post-translational modifications (phosphorylation and ubiquitination, respectively) important for assembly of B^act^ and the catalytically active B* complex (Figure S10).

### SAC3A

Sac3 proteins are evolutionarily conserved members of the **tr**anscription-**ex**port (TREX) complex, which was first defined in budding yeast as a complex coupling transcription to mRNA export from the nucleus (Strässer *et al.* 2002). In budding yeast, Sac3 is not an essential gene (Bauer and Kölling 1996) and, likewise, *SAC3A* is dispensable in *Arabidopsis*. The *sac3a* mutants we identified are viable, fertile, and generally appear indistinguishable from wild-type plants. A previous study also reported that a T-DNA insertion mutant of *sac3a* does not have a morphological mutant phenotype (Lu *et al.* 2010).

In *Arabidopsis*, SAC3A and another member of the SAC3 family, SAC3B, have been detected as constituents of the TREX-2 complex (Lu *et al.* 2010). Unexpectedly, however, triple (presumably) null mutations in *sac3a*, *sac3b*, and *sac3c* did not seem to impair mRNA transport (Lu *et al.* 2010). Confirming this finding requires more extensive examination of mRNA transport in the triple mutant, including tests of additional alleles in transport studies.

### Roles of *PRP4KA* and *SAC3A* in splicing

Although the splicing pattern of *GFP* pre-mRNA is not dramatically changed in *prp4ka-4* and *sac3a-6*, both mutants clearly exhibit reduced splicing efficiency of the noncanonical AT–AC intron in *GFP* pre-mRNA. This reduction likely contributes to the GFP-weak phenotypes of the mutants by decreasing the level of translatable *GFP* mRNA and, hence, GFP protein. The finding of diminished levels of the translatable AT–AC transcript, which results from splicing at splice sites that are less efficiently used by the U2-dependent spliceosome than canonical GT–AG splice sites (Crotti *et al.* 2007), is consistent with recent results from fission yeast showing that Prp4 kinase facilitates recognition of introns with weak splice sites (Eckert *et al.* 2016).

On a genome-wide scale, the *prp4ka* and *sac3a* mutants exhibit widespread perturbations in alternative splicing. These results demonstrate the functional relevance of *PRP4KA* and *SAC3A* for pre-mRNA splicing, a conclusion that is further supported by the overrepresentation of RNA-processing and splicing-related terms in the GO analyses of DEG and DAS genes in the two mutants as well as proteins undergoing phosphorylation changes in the *prp4ka* mutant. As expected, IR was the most frequently observed alternative splicing event, but an appreciable number of changes in other categories, particularly alternative 5′ and 3′ splice selection, was also detected. The overlap between the DEGs and DAS events in the two mutants was only partial despite their similar patterns of *GFP* pre-mRNA splicing and high levels of coexpression, which suggested that they may function in the same process (Usadel *et al.* 2009). These findings reinforce the complex nature of alternative splicing and are in accord with earlier findings in budding yeast that mutations in given splicing factors have quite different effects on individual genes (Pleiss *et al.* 2007).

The genome-wide analysis of introns more retained in *prp4ka* and *sac3a* mutants revealed a tendency toward somewhat weaker 5′ splice sites and an increased GC content. Alternatively regulated introns common to both the *prp4ka* and *sac3a* mutants are significantly longer than the introns regulated in either of the single mutants. Strikingly, ∼42% of the more-retained introns in the *prp4ka* mutant were found to be first introns. The exact role of PRP4KA in the splicing of first introns remains to be determined.

### Potential substrates of PRP4KA

Prp4 kinase in fission yeast phosphorylates the SR protein Srp2 (Lützelberger and Käufer 2012) and in human cells the splicing factors Prp6 (STA1, At4g43030 in *Arabidopsis*) and Prp31 (PRP31A, At1g60170 in *Arabidopsis*) during formation of the catalytically active B* complex (Schneider *et al.* 2010). We did not detect the *Arabidopsis* orthologs of these proteins in the iTRAQ analysis of the *prp4ka* mutant. However, our findings are generally in agreement with the previous studies in that we identified 5 SR proteins and 15 other splicing-related factors that change in phosphorylation level in the *prp4ka* mutant. Ten of these splicing-related proteins significantly lose phosphorylation and hence are potentially direct substrates of PRP4KA activity. A number of additional RNA-binding proteins not yet implicated in splicing similarly lose phosphorylation in the *prp4ka* mutant, suggesting they may also be directly targeted for phosphorylation by PRP4KA.

Splicing factors and other proteins that gain phosphorylation in the *prp4ka* mutant are presumably responding indirectly to a reduction in PRP4KA activity, perhaps through another protein kinase or phosphatase that is itself modified by PRP4KA. Potential phosphorylation substrates of nonsplicing factors that were identified in the iTRAQ analysis could reflect additional roles for PRP4KA. For example, Prp4k in human cells has been implicated in coupling pre-mRNA splicing with chromatin remodeling events that regulate transcription (Dellaire *et al.* 2002) and in mitosis (Montembault *et al.* 2007).

Some of the phosphorylated residues we identified in SR proteins and other splicing-related factors were also detected in a previous phosphoproteomic analysis of proteins involved in RNA metabolism in *Arabidopsis* (de la Fuente van Bentem *et al.* 2006). In the prior study, it was noted that phosphorylation in splicing factors often occurs at a serine or threonine followed by a proline (pSP or pTP). We observed a similar trend, suggesting that PRP4KA may frequently target SP and TP sites.

### General comments and speculation

As discussed above, *PRP4KA* and *SAC3A* are highly coexpressed with each other, and the respective mutants have similar GFP-weak phenotypes and patterns of *GFP* pre-mRNA splicing. These observations drew our attention to the possibility of a novel functional relationship between the two proteins. Whereas splicing regulation is a recognized function of Prp4 kinases, Sac3 proteins have not been directly associated with splicing but rather with the aforementioned role in mRNA export. However, given the known coupling between transcription and splicing (Naftelberg *et al.* 2015), and the subsequent requirement to export mature mRNAs out of the nucleus, it is conceivable that PRP4KA and SAC3A cooperate during the transition between these consecutive processes. In fission yeast, Prp4k has been proposed to act as a checkpoint kinase that only permits properly spliced transcripts to exit the nucleus (Lützelberger and Käufer 2012). Extrapolating from this suggestion, it is conceivable that PRP4KA and SAC3A cooperate functionally to link splicing quality control and nuclear export. Under this hypothesis, the *prp4ka* and *sac3a* mutations would affect not only splicing of *GFP* pre-mRNA but also retard the efflux of mature *GFP* mRNA from the nucleus to the cytoplasm. Both of these deficiencies would contribute additively to the GFP-weak phenotype of the mutants. In this context, it is interesting to note that the iTRAQ analysis identified several nuclear pore and nuclear transport proteins as potential substrates of PRP4KA. Clearly, further work is required to understand the functional relationship between PRP4KA and SAC3A, and to define potentially expanded roles for these proteins. The *prp4ka* and *sac3a* mutants we identified and the easily monitored alternatively spliced *GFP* reporter gene system should be useful tools for these investigations.

## References

[bib1] Addepalli B. Hunt A. G., 2008 Ribonuclease activity is a common property of Arabidopsis CCCH-containing zinc-finger proteins. FEBS Lett. 582: 2577–2582. 10.1016/j.febslet.2008.06.029 18582464

[bib2] Alahari S. K. Schmidt H. Käufer N. F., 1993 The fission yeast prp4+ gene involved in pre-mRNA splicing codes for a predicted serine/threonine kinase and is essential for growth. Nucleic Acids Res. 21: 4079–4083. 10.1093/nar/21.17.4079 8371982 PMC310008

[bib3] Alamancos G. P. Pagès A. Trincado J. L. Bellora N. Eyras E., 2015 Leveraging transcript quantification for fast computation of alternative splicing profiles. RNA 21: 1521–1531. 10.1261/rna.051557.115 26179515 PMC4536314

[bib4] Al-Ayoubi A. M. Zheng H. Liu Y. Bai T. Eblen S. T., 2012 Mitogen-activated protein kinase phosphorylation of splicing factor 45 (SPF45) regulates SPF45 alternative splicing site utilization, proliferation, and cell adhesion. Mol. Cell. Biol. 32: 2880–2893. 10.1128/MCB.06327-11 22615491 PMC3416182

[bib6] Baralle M. M. Baralle F. E., 2018 The splicing code. Biosystems 164: 39–48. 10.1016/j.biosystems.2017.11.002 29122587

[bib7] Barash Y. Calarco J. A. Gao W. Pan Q. Wang X., 2010 Deciphering the splicing code. Nature 465: 53–59. 10.1038/nature09000 20445623

[bib8] Barta A. Kalyna M. Lorković Z. J., 2008 Plant SR proteins and their functions. Curr. Top. Microbiol. Immunol. 326: 83–102. 18630748 10.1007/978-3-540-76776-3_5

[bib9] Barta A. Kalyna M. Reddy A. S., 2010 Implementing a rational and consistent nomenclature for serine/arginine-rich protein splicing factors (SR proteins) in plants. Plant Cell 22: 2926–2929. 10.1105/tpc.110.078352 20884799 PMC2965536

[bib10] Bauer A. Kölling R., 1996 Characterization of the SAC3 gene of *Saccharomyces cerevisiae*. Yeast 12: 965–975. 10.1002/(SICI)1097-0061(199608)12:10<965::AID-YEA999>3.0.CO;2-Q 8873450

[bib11] Boesler C. Rigo N. Anokhina M. M. Tauchert M. J. Agafonov D. E., 2016 A spliceosome intermediate with loosely associated tri-snRNP accumulates in the absence of Prp28 ATPase activity. Nat. Commun. 7: 11997 10.1038/ncomms11997 27377154 PMC4935976

[bib12] Bottner C. A. Schmidt H. Vogel S. Michele M. Käufer N. F., 2005 Multiple genetic and biochemical interactions of Brr2, Prp8, Prp31, Prp1 and Prp4 kinase suggest a function in the control of the activation of spliceosomes in *Schizosaccharomyces pombe*. Curr. Genet. 48: 151–161. 10.1007/s00294-005-0013-6 16133344

[bib13] Braunschweig U. Barbosa-Morais N. L. Pan Q. Nachman E. N. Alipanahi B., 2014 Widespread intron retention in mammals functionally tunes transcriptomes. Genome Res. 24: 1774–1786. 10.1101/gr.177790.114 25258385 PMC4216919

[bib14] Burge C. B. Padgett R. A. Sharp P. A., 1998 Evolutionary fates and origins of U12-type introns. Mol. Cell 2: 773–785. 10.1016/S1097-2765(00)80292-0 9885565

[bib78] Clough S. J. Bent A. F., 1998 Floral dip: a simplified method for *Agrobacterium*-mediated transformation of *Arabidopsis thaliana*. Plant J. 16: 735–743. 10069079 10.1046/j.1365-313x.1998.00343.x

[bib15] Crotti L. B. Bacíková D. Horowitz D., 2007 The Prp18 protein stabilizes the interaction of both exons with the U5 snRNA during the second step of pre-mRNA splicing. Genes Dev. 21: 1204–1216. 10.1101/gad.1538207 17504938 PMC1865492

[bib16] de la Fuente van Bentem S. Anrather D. Roitinger E. Djamei A. Hufnagl T., 2006 Phosphoproteomics reveals extensive in vivo phosphorylation of Arabidopsis proteins involved in RNA metabolism. Nucleic Acids Res. 34: 3267–3278. 10.1093/nar/gkl429 16807317 PMC1904105

[bib17] de la Fuente van Bentem S. Anrather D. Dohnal I. Roitinger E. Csaszar E., 2008 Site-specific phosphorylation profiling of Arabidopsis proteins by mass spectrometry and peptide chip analysis. J. Proteome Res. 7: 2458–2470. 10.1021/pr8000173 18433157

[bib18] Dellaire G. Makarov E. M. Cowger J. J. Longman D. Sutherland H. G., 2002 Mammalian PRP4 kinase copurifies and interacts with components of both the U5 snRNP and the N-CoR deacetylase complexes. Mol. Cell. Biol. 22: 5141–5156. 10.1128/MCB.22.14.5141-5156.2002 12077342 PMC139773

[bib19] Eckert D. Andrée N. Razanau A. Zock-Emmenthal S. Lützelberger M., 2016 Prp4 kinase grants the license to splice: control of weak splice sites during spliceosome activation. PLoS Genet. 12: e1005768 10.1371/journal.pgen.1005768 26730850 PMC4701394

[bib20] Fair B. J. Pleiss J. A., 2017 The power of fission: yeast as a tool for understanding complex splicing. Curr. Genet. 63: 375–380. 10.1007/s00294-016-0647-6 27628706 PMC5350050

[bib21] Feilner T. Hultschig C. Lee J. Meyer S. Immink R. G., 2005 High throughput identification of potential Arabidopsis mitogen-activated protein kinases substrates. Mol. Cell. Proteomics 4: 1558–1568. 10.1074/mcp.M500007-MCP200 16009969

[bib22] Filichkin S. Priest H. D. Megraw M. Mockler T. C., 2015 Alternative splicing in plants: directing traffic at the crossroads of adaptation and environmental stress. Curr. Opin. Plant Biol. 24: 125–135. 10.1016/j.pbi.2015.02.008 25835141

[bib23] Fluhr R., 2008 Regulation of splicing by protein phosphorylation. Curr. Top. Microbiol. Immunol. 326: 119–138. 18630750 10.1007/978-3-540-76776-3_7

[bib24] Fu J. L. Kanno T. Liang S. C. Matzke A. J. Matzke M., 2015 GFP loss-of-function mutations in *Arabidopsis thaliana*. G3 (Bethesda) 5: 1849–1855. 10.1534/g3.115.019604 26153075 PMC4555221

[bib25] Gao X. Jin Q. Jiang C. Li Y. Li C., 2016 FgPrp4 kinase is important for spliceosome B-complex activation and splicing efficiency in *Fusarium graminearum*. PLoS Genet. 12: e1005973 10.1371/journal.pgen.1005973 27058959 PMC4825928

[bib26] Golovkin M. Reddy A. S., 1999 An SC35-like protein and a novel serine/arginine-rich protein interact with Arabidopsis U1–70K protein. J. Biol. Chem. 274: 36428–36438. 10.1074/jbc.274.51.36428 10593939

[bib27] Gould G. M. Paggi J. M. Guo Y. Phizicky D. V. Zinshteyn B., 2016 Identification of new branch points and unconventional introns in *Saccharomyces cerevisiae*. RNA 22: 1522–1534. 10.1261/rna.057216.116 27473169 PMC5029451

[bib28] Higa L. A. Wu M. Ye T. Kobayashi R. Sun H., 2006 CUL4–DDB1 ubiquitin ligase interacts with multiple WD40-repeat proteins and regulates histone methylation. Nat. Cell Biol. 8: 1277–1283. 10.1038/ncb1490 17041588

[bib29] James G. V. Patel V. Nordström K. J. Klasen J. R. Salomé P. A., 2013 User guide for mapping-by-sequencing in *Arabidopsis*. Genome Biol. 14: R61 10.1186/gb-2013-14-6-r61 23773572 PMC3706810

[bib30] Kanno T. Bucher E. Daxinger L. Huettel B. Böhmdorfer G., 2008 A structural-maintenance-of-chromosomes hinge domain-containing protein is required for RNA-directed DNA methylation. Nat. Genet. 40: 670–675. 10.1038/ng.119 18425128

[bib31] Kanno T. Lin W. D. Fu J. L. Wu M. T. Yang H. W., 2016 Identification of coilin mutants in a screen for enhanced expression of an alternatively spliced GFP reporter gene in *Arabidopsis thaliana*. Genetics 203: 1709–1720. 10.1534/genetics.116.190751 27317682 PMC4981272

[bib32] Kanno T. Lin W. D. Fu J. L. Matzke A. J. M. Matzke M., 2017a A genetic screen implicates a CWC16/Yju2/CCDC130 protein and SMU1 in alternative splicing in *Arabidopsis thaliana*. RNA 23: 1068–1079. 10.1261/rna.060517.116 28373290 PMC5473141

[bib33] Kanno T. Lin W. D. Fu J. L. Chang C. L. Matzke A. J. M., 2017b A genetic screen for pre-mRNA splicing mutants of *Arabidopsis thaliana* identifies putative U1 snRNP components RBM25 and PRP39a. Genetics 207: 1347–1359. 10.1534/genetics.117.300149 28971960 PMC5714452

[bib34] Kanno T. Lin W. D. Chang C. L. Matzke M. Matzke A. J. M., 2018 A genetic screen identifies PRP18a, a putative second step splicing factor important for alternative splicing and a normal phenotype in Arabidopsis thaliana. G3 (Bethesda) 8: 1367–1377. 10.1534/g3.118.200022 29487188 PMC5873924

[bib79] Kim Y. Schumaker K. S. Zhu J.K. 2006 EMS mutagenesis of *Arabidopsis*. Methods Mol. Biol. 323: 101–103. 16739570 10.1385/1-59745-003-0:101

[bib35] Koncz C. Dejong F. Villacorta N. Szakonyi D. Koncz Z., 2012 The spliceosome-activating complex: molecular mechanisms underlying the function of a pleiotropic regulator. Front. Plant Sci. 3: 9 10.3389/fpls.2012.00009 22639636 PMC3355604

[bib36] Koroleva O. A. Tomlinson M. L. Leader D. Shaw P. Doonan J. H., 2005 High-throughput protein localization in Arabidopsis using Agrobacterium-mediated transient expression of GFP-ORF fusions. Plant J. 41: 162–174. 10.1111/j.1365-313X.2004.02281.x 15610358

[bib37] Lan P. Li W. Wen T. N. Shiau Y. S. Wu Y. C., 2011 iTRAQ protein profile analysis of Arabidopsis roots reveals new aspects critical for iron homeostasis. Plant Physiol. 155: 821–834. 10.1104/pp.110.169508 21173025 PMC3032469

[bib38] Lehti-Shiu M. D. Shiu S. H., 2012 Diversity, classification and function of the plant protein kinase superfamily. Philos. Trans. R. Soc. Lond. B Biol. Sci. 367: 2619–2639. 10.1098/rstb.2012.0003 22889912 PMC3415837

[bib39] Li, H., 2013 Aligning sequence reads, clone sequences and assembly contigs with BWA-MEM. arXiv:1303.3997v1 [q-bio.GN].

[bib40] Lu Q. Tang X. Tian G. Wang F. Liu K., 2010 Arabidopsis homolog of the yeast TREX-2 mRNA export complex: components and anchoring nucleoporin. Plant J. 61: 259–270. 10.1111/j.1365-313X.2009.04048.x 19843313

[bib41] Lützelberger M. Käufer N. F., 2012 The Prp4 kinase: its substrates, function and regulation in pre-mRNA splicing in Protein Phosphorylation in Human Health. InTechOpen, London Available at: http://www.intechopen.com/books/protein-phosphorylation-inhuman-Health.

[bib42] Marquez Y. Brown J. W. Simpson C. Barta A. Kalyna M., 2012 Transcriptome survey reveals increased complexity of the alternative splicing landscape in *Arabidopsis*. Genome Res. 22: 1184–1195. 10.1101/gr.134106.111 22391557 PMC3371709

[bib43] Marquez Y. Höpfler M. Ayatollahi Z. Barta A. Kalyna M., 2015 Unmasking alternative splicing inside protein-coding exons defines exitrons and their role in proteome plasticity. Genome Res. 25: 995–1007. 10.1101/gr.186585.114 25934563 PMC4484396

[bib44] Matera A. G. Wang Z., 2014 A day in the life of the spliceosome. Nat. Rev. Mol. Cell Biol. 15: 108–121 (erratum: Nat. Rev. Mol. Cell Biol. 15: 294) 10.1038/nrm3742 24452469 PMC4060434

[bib45] Matzke A. J. Watanabe K. van der Winden J. Naumann U. Matzke M., 2010 High frequency, cell type-specific visualization of fluorescent-tagged genomic sites in interphase and mitotic cells of living *Arabidopsis* plants. Plant Methods 6: 2 10.1186/1746-4811-6-2 20148117 PMC2820019

[bib46] Meyer F., 2016 Viral interactions with components of the splicing machinery. Prog. Mol. Biol. Transl. Sci. 142: 241–268. 10.1016/bs.pmbts.2016.05.008 27571697

[bib47] Montembault E. Dutertre S. Prigent C. Giet R., 2007 PRP4 is a spindle assembly checkpoint protein required for MPS1, MAD1, and MAD2 localization to the kinetochores. J. Cell Biol. 179: 601–609. 10.1083/jcb.200703133 17998396 PMC2080909

[bib48] Naftelberg S. Schor I. E. Ast G. Kornblihtt A. R., 2015 Regulation of alternative splicing through coupling with transcription and chromatin structure. Annu. Rev. Biochem. 84: 165–198. 10.1146/annurev-biochem-060614-034242 26034889

[bib50] Nilsen T. W. Graveley B. R., 2010 Expansion of the eukaryotic proteome by alternative splicing. Nature 463: 457–463. 10.1038/nature08909 20110989 PMC3443858

[bib51] Novick P. Osmond B. C. Botstein D., 1989 Suppressors of yeast actin mutations. Genetics 121: 659–674. 2656401 10.1093/genetics/121.4.659 PMC1203651

[bib52] Patro R. Duggal G. Love M. I. Irizarry R. A. Kingsford C., 2017 Salmon provides fast and bias-aware quantification of transcript expression. Nat. Methods 14: 417–419. 10.1038/nmeth.4197 28263959 PMC5600148

[bib80] Pietrzak M. Shillito R. D. Hohn T. Potrykus I., 1986 Expression in plants of two bacterial antibiotic resistance genes after protoplast transformation with a new plant expression vector. Nucleic Acids Res. 14: 5857–5868. 3016666 10.1093/nar/14.14.5857 PMC311596

[bib53] Pleiss J. A. Whitworth G. B. Bergkessel M. Guthrie C., 2007 Transcript specificity in yeast pre-mRNA splicing revealed by mutations in core spliceosomal components. PLoS Biol. 5: e90 10.1371/journal.pbio.0050090 17388687 PMC1831718

[bib54] Pozzi B. Bragado L. Will C. L. Mammi P. Risso G., 2017 SUMO conjugation to spliceosomal proteins is required for efficient pre-mRNA splicing. Nucleic Acids Res. 45: 6729–6745. 10.1093/nar/gkx213 28379520 PMC5499870

[bib55] Robinson M. D. McCarthy D. J. Smyth G. K., 2010 edgeR: a Bioconductor package for differential expression analysis of digital gene expression data. Bioinformatics 26: 139–140. 10.1093/bioinformatics/btp616 19910308 PMC2796818

[bib56] Rosembert, M., 2017 The role of pre-mRNA splicing and splicing-related proteins in the cold acclimation induced adjustment of photosynthesis and the acquisition of freezing tolerance in Arabidopsis thaliana. Ph.D. Dissertation, University of Ottawa, Ottawa.

[bib57] Sasaki T. Kanno T. Liang S. C. Chen P. Y. Liao W. W., 2015 An Rtf2 domain-containing protein influences pre-mRNA splicing and is essential for embryonic development in *Arabidopsis thaliana*. Genetics 200: 523–535. 10.1534/genetics.115.176438 25819795 PMC4492377

[bib58] Savaldi-Goldstein S. Sessa G. Fluhr R., 2000 The ethylene-inducible PK12 kinase mediates the phosphorylation of SR splicing factors. Plant J. 21: 91–96. 10.1046/j.1365-313x.2000.00657.x 10652154

[bib59] Schneider M. Hsiao H. H. Will C. L. Giet R. Urlaub H., 2010 Human PRP4 kinase is required for stable tri-snRNP association during spliceosomal B complex formation. Nat. Struct. Mol. Biol. 17: 216–221. 10.1038/nsmb.1718 20118938

[bib60] Sheth N. Roca X. Hastings M. L. Roeder T. Krainer A. R., 2006 Comprehensive splice-site analysis using comparative genomics. Nucleic Acids Res. 34: 3955–3967. 10.1093/nar/gkl556 16914448 PMC1557818

[bib61] Sibley C. R. Blazquez L. Ule J., 2016 Lessons from non-canonical splicing. Nat. Rev. Genet. 17: 407–421. 10.1038/nrg.2016.46 27240813 PMC5154377

[bib62] Soneson C. Love M. I. Robinson M. D., 2016 Differential analyses for RNA-seq: transcript-level estimates improve gene-level inferences. F1000 Res. 4: 1521 10.12688/f1000research.7563.2 PMC4712774 26925227

[bib63] Staiger D. Brown J. W., 2013 Alternative splicing at the intersection of biological timing, development, and stress responses. Plant Cell 25: 3640–3656. 10.1105/tpc.113.113803 24179132 PMC3877812

[bib64] Staiger D. Simpson G. G., 2015 Enter exitrons. Genome Biol. 16: 136 10.1186/s13059-015-0704-3 26149172 PMC4494720

[bib65] Stamm S., 2008 Regulation of alternative splicing by reversible protein phosphorylation. J. Biol. Chem. 283: 1223–1227. 10.1074/jbc.R700034200 18024427

[bib66] Strässer K. Masuda S. Mason P. Pfannstiel J. Oppizzi M., 2002 TREX is a conserved complex coupling transcription with messenger RNA export. Nature 417: 304–308. 10.1038/nature746 11979277

[bib67] Szakonyi D. Duque P., 2018 Alternative splicing as a regulator of early plant development. Front. Plant Sci. 9: 1174 10.3389/fpls.2018.01174 30158945 PMC6104592

[bib68] Thomas P. D. Campbell M. J. Kejariwal A. Mi H. Karlak B., 2003 PANTHER: a library of protein families and subfamilies indexed by function. Genome Res. 13: 2129–2141. 10.1101/gr.772403 12952881 PMC403709

[bib69] Turunen J. J. Niemelä E. H. Verma B. Frilander M. J., 2013 The significant other: splicing by the minor spliceosome. Wiley Interdiscip. Rev. RNA 4: 61–76. 10.1002/wrna.1141 23074130 PMC3584512

[bib70] Usadel B. Obayashi T. Mutwil M. Giorgi F. M. Bassel G. W., 2009 Co-expression tools for plant biology: opportunities for hypothesis generation and caveats. Plant Cell Environ. 32: 1633–1651. 10.1111/j.1365-3040.2009.02040.x 19712066

[bib71] Van der Auwera G. A. Carneiro M. O. Hartl C. Poplin R. Del Angel G., 2013 From FastQ data to high confidence variant calls: the genome analysis toolkit best practices pipeline. Curr. Protoc. Bioinformatics 43: 11.10.1–11.10.33. 10.1002/0471250953.bi1110s43 25431634 PMC4243306

[bib72] Vélez-Bermúdez I. C. Wen T. N. Lan P. Schmidt W., 2016 Isobaric tag for relative and absolute quantitation (iTRAQ)-based protein profiling in plants. Methods Mol. Biol. 1450: 213–221. 10.1007/978-1-4939-3759-2_17 27424757

[bib73] Vizcaíno J. A. Csordas A. del-Toro N. Dianes J. A. Griss J., 2016 2016 update of the PRIDE database and its related tools. Nucleic Acids Res. 44: D447–D456 (erratum: Nucleic Acids Res. 44: 11033) 10.1093/nar/gkv1145 26527722 PMC4702828

[bib74] Wahl M. C. Lührmann R., 2015 SnapShot: spliceosome dynamics I. Cell 161: 1474-e1 10.1016/j.cell.2015.05.050 26046445

[bib75] Will C. L. Lührmann R., 2011 Spliceosome structure and function. Cold Spring Harb. Perspect. Biol. 3: a003707 10.1101/cshperspect.a003707 21441581 PMC3119917

[bib76] Zhang R. Calixto C. P. G. Marquez Y. Venhuizen P. Tzioutziou N. A., 2017 A high quality Arabidopsis transcriptome for accurate transcript-level analysis of alternative splicing. Nucleic Acids Res. 45: 5061–5073. 10.1093/nar/gkx267 28402429 PMC5435985

[bib77] Zhou Z. Fu X. D., 2013 Regulation of splicing by SR proteins and SR protein-specific kinases. Chromosoma 122: 191–207. 10.1007/s00412-013-0407-z 23525660 PMC3660409

